# Thermal transport through molecular monolayers in plasmonic nanogaps

**DOI:** 10.1038/s41467-026-73256-0

**Published:** 2026-05-22

**Authors:** Fiona Bell, Erfan Norouzi Farahani, Yeeun Roh, Sara Sangtarash, Zhenyao Jiang, Hatef Sadeghi, Jeremy J. Baumberg

**Affiliations:** 1 NanoPhotonics Centre, Cavendish Laboratory, Department of Physics, JJ Thompson Avenue, University of Cambridge, Cambridge, UK; 2 Quantum Device Modelling Group, School of Engineering, University of Warwick, Coventry, UK

**Keywords:** Organic-inorganic nanostructures, Nanophotonics and plasmonics, Molecular electronics

## Abstract

Thermal transport through molecules has been exceptionally difficult to measure robustly. This is despite many intriguing theoretical predictions such as thermal diodes or gated thermal conduction, with consequent useful applications. Here, we present a significant development in time-resolved photothermal measurements using the extreme plasmonic enhancement of light inside molecular nanogaps to follow thermal transport on the nanoscale. We exploit free-standing sheets of close-packed nanoparticles, which are locally heated using mid-infrared pulses, and track their cooling by lateral transport on microsecond timescales. The rate of thermal relaxation is found to be dependent on nanogap composition, measured for a series of thiolated aromatic molecules. Observed trends in thermal conductivity with molecular length and contact strength are broadly reproduced by non-equilibrium molecular dynamics simulations. However, we find additional consideration of molecular interactions and dynamic disorder are crucial in fully understanding our experimental findings.

## Abstract (web summary)

Thermal conduction of single molecule layers has been extremely difficult to measure. Here, the authors use a transient optical technique on precision-assembled nanostructures to characterise different molecular spacers.

## Introduction

The fundamental properties of heat transport through molecules have been of interest for many years, but because of their strong dependence on conformation, small length, and the significant influence of contacting, it has remained extremely difficult to measure^1–3^. So far, only a few molecules, including alkanes, oligo(phenylene-ethynylene)_3_ (OPE3), and anthracene, have been measured at the single-molecule level^3–7^. This is despite increasing theoretical interest, which has demonstrated the possibility of manipulating heat transport through molecular materials by engineering their chemical structure. Applications range from thermal diode and switching behaviours^8^ to thermal management^9^ and thermoelectric energy conversion^10–13^. The latter requires low thermal conductance, which molecular materials offer. In these materials, effects such as phonon interference^14–18^ and mismatches at the interfaces^19–21^ can be used to further control heat flow. Composite nanomaterials combining inorganic and organic components interleaved at near thermal length scales are even less explored, with phononic contributions to thermal conductance varying due to their distinct Debye energies^22^.

The capability to orient molecular monolayers in nanoscale gaps has transformed the field of molecular electronics, with many charge transport capabilities from inorganic systems now duplicated and well matched to theory. However, corresponding studies of thermal transport remain at a much earlier stage, owing to the difficulty of measuring heat flows on the nanometre scale. While macroscopic measurements (for instance, of polymers) are well explored, morphologies comprising molecular surface-assembled monolayers (SAMs) have been challenging to measure, especially in single junctions. In particular, the heterogeneity of multiple junctions confounds systematic results, as does the formation of different single-molecule conformations when dynamic pull-off experiments are performed by STM^4,23,24^.

In this work, we present an ensemble approach to measuring thermal transport within an interconnected network of $\sim$500 well-defined molecular nanogaps. A 1D chain of nanoparticles (NPs) presents the simplest possible geometry but proves hard to achieve consistently. Instead, we study 2D near-monolayer sheets of NPs^25–29^ (MLaggs), which can be similarly analysed and are more resilient against disorder. Identical metal-molecule nano-junctions are assembled in MLaggs of close-packed AuNPs, which are each thermally connected by a SAM of thiolated aromatic molecules. An optical technique is used to heat and measure local temperature dynamics, following the in-plane lateral thermal flow between neighbouring NPs in the MLagg. Careful comparison between a sequence of SAM molecules reveals trends within their thermal conductivity. We discuss the mechanisms that control this transport using molecular dynamics simulations, including the effects of molecular interactions and binding with the metal facets.

## Results and discussion

**Fig. 1:**
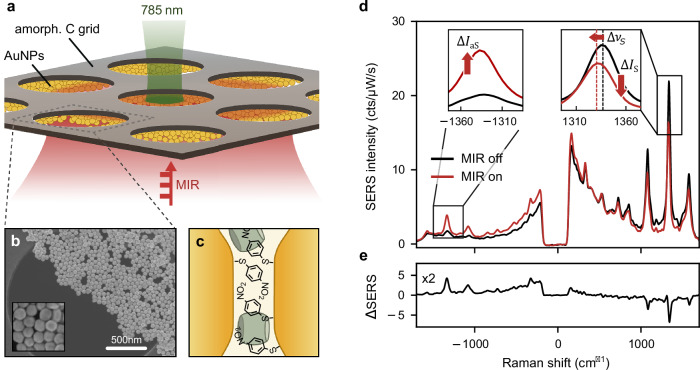
MIR-induced modulation of SERS from an s-MLagg. **a** AuNP monolayer aggregate suspended on an amorphous C grid (hole diameter 2 µm) pumped by pulsed MIR and 785 nm probe. **b** SEM image of suspended MLagg edge. Inset: further 3$\times$ magnification. **c** Illustration of a nanogap formed by binding 60 nm AuNPs with a CB[5] spacer (green) and near-complete replacement by NTP. **d**, **e** SERS spectra from NTP-filled s-MLagg showing (**d**) raw modulation and (**e**) induced SERS changes ($\Delta$SERS) under MIR illumination tuned to 1335 cm^‒1^ (box). Insets in (**d**) highlight intensity modulation of NTP nitro-stretch vibration at Stokes ($\Delta {I}_{{{{\rm{S}}}}}$) and antiStokes ($\Delta {I}_{{{{\rm{aS}}}}}$) frequencies along with Stokes frequency redshift ${\Delta \nu }_{s}$. Intensities at negative wavenumbers (aS) in (**e**) multiplied by x2.

MLaggs are formed by depositing a monolayer film of precision-spaced 60 nm AuNPs onto a 20nm-thick amorphous carbon grid (Fig. 1a, b). Nanogaps of 0.9 nm width are initially defined by a cucurbituril molecular spacer (CB[5])^28,30^, which strongly adheres to AuNPs and delivers a Young's modulus sufficient to support a suspended MLagg film (s-MLagg) across 2 µm diameter holes in the amorphous C film. The s-MLagg is subsequently immersed in a molecular solution of 4-nitrothiophenol (NTP), and near-complete replacement of CB[5] is confirmed by both Fourier Transform Infrared (FTIR) and Raman spectroscopy (Fig. 1c and Supplementary Note 2). Using co-aligned objective lenses, samples are first excited from below by a pulsed MIR laser while a 785 nm CW laser incident from above collects surface-enhanced Raman scattering (SERS) in back-reflection. The tight field confinement in the nanogaps and efficient optical coupling mean that SERS is both extremely consistent and strongly enhanced in MLaggs^26,31–33^, allowing temperatures to be measured in-situ. Away from any supporting substrate, plasmonic nanogaps in regions of the suspended MLagg are directly accessible by both optical beams with excitation intensities of MIR (50 µW/µm^2^) and near-infrared at 785 nm (30 µW/µm^2^) efficiently in-coupled at diffraction-limited spots.

The typical CW SERS spectrum of an s-MLagg (Fig. 1d) shows a series of sharp Raman-active vibrational peaks from NTP, superimposed on a background of electronic scattering from the Au. Weak Raman-active vibrations of CB[5] are not visible. MIR illumination at frequency ${\nu }_{{{{\rm{MIR}}}}}=$ 1335 cm^‒1^ induces a strong intensity modulation of scattered light (arrows, Fig. 1d, e), similar to previously reported measurements of individual plasmonic nanojunctions^34,35^. The intensity of both vibrational and electronic SERS signals is observed to decrease for positive Raman shifts (Stokes scattering) and increase for negative Raman shifts (antiStokes scattering). Additionally, the frequency of all vibrational modes is found to undergo a red shift on the order of a few wavenumbers.

**Fig. 2:**
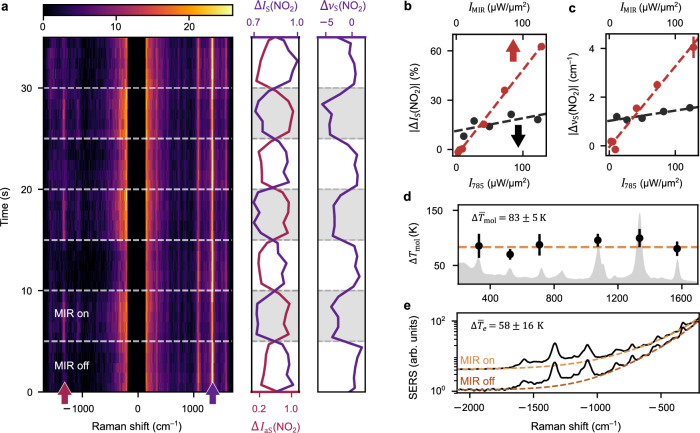
MIR-induced temperature changes of NTP:CB[5] MLagg. **a** SERS spectra over time, with MIR alternating off/on every 5 spectra. Insets (right) track modulation of Stokes and antiStokes NO_2_ peaks (arrows on timescan). Intensity normalised to initial MIR-off spectrum. **b**, **c** 785 nm (black) and MIR (red) power dependence of SERS modulation measured by average **b** Stokes intensity change $\Delta {I}_{S}({{{\rm{N}}}}{{{{\rm{O}}}}}_{2})$ and **c** Stokes frequency shift $\Delta {\nu }_{S}({{{\rm{N}}}}{{{{\rm{O}}}}}_{2})$ of NO_2_ vibration. Corresponding constant powers are ${I}_{{{{\rm{MIR}}}}}$ = 50 µW/µm^2^ and ${I}_{785}$ = 30 µW/µm^2^. **d**, **e** Estimated MIR-induc**e**d (**d**) molecular temperature change $\Delta {\bar{T}}_{{{{\rm{mol}}}}},$ and **e** electronic temperature change $\Delta {\bar{T}}_{{{{\rm{e}}}}}$. For both, the average temperature over 4 locations is shown. Average on and off spectra given in (**e**).

Modulation of both the SERS intensities and the frequency of vibrational modes is fully reversible, demonstrating no visible damage over multiple cycles of MIR illumination (Fig. 2a). Analysis of the NTP NO_2_ vibrational stretch at 1335 cm^–1^ shows a step-like modulation of SERS intensity upon MIR illumination, with a 30% decrease of Stokes intensity $\Delta {I}_{{{{\rm{S}}}}}({{{{\rm{NO}}}}}_{2})$ and a 400% increase of antiStokes intensity $\Delta {I}_{{{{\rm{aS}}}}}({{{{\rm{NO}}}}}_{2})$. A reproducible frequency shift ${\Delta \nu }_{S}({{{{\rm{NO}}}}}_{2})=$ –5 cm^–1^ is seen, and is similar for the antiStokes side (though less well resolved due to the weak signal). This magnitude of SERS modulation shows a strong MIR power-dependence, with amplitudes of both $|\Delta {I}_{{{{\rm{S}}}}}({{{{\rm{NO}}}}}_{2})|$ and $|{\Delta \nu }_{S}({{{{\rm{NO}}}}}_{2})|$ linearly increasing with MIR power density (red, Fig. 2b, c). By contrast, the 785 nm power has little effect on the strength of SERS modulation (black, Fig. 2b, c). The observed perturbation of SERS signatures is therefore primarily the result of MIR-induced changes in the sample.

### MIR-induced temperature change

Increased antiStokes emission has previously been observed within similar plasmonic systems, attributed to a wide range of optically-induced phenomena such as inelastic electron tunnelling^36^, molecular frequency up-conversion^37–39^, vibrational pumping^40^, and the formation of picocavities^41–43^. However, in the s-MLagg, the presence of additional MIR-induced effects (namely a vibrational frequency shift and modulation of electronic background) indicates that changes in SERS scattering are of thermal origin, induced by a local MIR-heating effect. This is further evidenced by the sensitivity of SERS modulation to the substrate material: the equivalent measurements for MLaggs placed on a CaF_2_ substrate show minimal modulation under MIR illumination (Supplementary Fig. 2). In comparison to thin amorphous C, crystalline CaF_2_ supports a large bath of thermal phonons to which excess heat is rapidly dissipated. Isolation of the plasmonic system is therefore crucial for observing these photothermal effects.

The primary reason for identifying this as a thermal mechanism is the rise and decay time-scales involved. MIR up-conversion disappears within a few ps, as do vibrational pumping and optomechanical effects, because the associated vibrational lifetimes are less than a few ps^44^. Inelastic tunnelling of electrons could only persist for µs if charge is redistributed by the MIR between nanoparticles and slowly tunnels back, but this would not explain the similar electronic heating observed, nor the vibrational shifts. Measured electronic conductivities of the MLagg are <10^−5^ S, while single molecule conductances of <10^−6^ S here^45^ imply <10 pW/K electron contributions, which are orders of magnitude lower than the typical phonon thermal conductance in such systems, further ruling out electronic contributions. Finally, picocavity decay in these nanogaps is much slower^41,42^, often >1 s, and not linear in power as observed for the signals here. We also note that all such electronic or optomechanical processes would be expected to also operate for MLaggs on a CaF_2_ substrate, in disagreement with our findings.

Within a classical model of Raman scattering, the antiStokes intensity ${I}_{{{{\rm{aS}}}}}$ is directly proportional to the phonon occupation ${n}_{v}$^46^, which increases upon heating to a non-equilibirum thermal state. High wavenumber vibrations >500 cm^–1^, with negligible thermal occupation at room temperature, are expected to display the greatest percentage increase of ${I}_{{{{\rm{aS}}}}}$. This is indeed reflected in the MIR-modulated spectra shown in Fig. 1d. The frequency shift of vibrational modes is also attributed to MIR-induced heating of molecules^47^. Low-wavenumber ro-vibrational modes, highly sensitive to temperature, modify the energy of high-frequency vibrations through anharmonic inter-mode coupling. Consequently, molecules exhibit a temperature-dependent frequency shift dictated by the coupling of individual vibrational modes, previously reported in SERS^48–50^. Increased background antiStokes electronic scattering is also consistent with a local temperature increase^51^. This Au electronic scattering is well described by a model considering the inelastic scattering of light from an electron gas^52^, with intensity dependent on the bosonic thermal population, which follows a Bose-Einstein distribution.

Based on this, the MIR-induced change of both molecular and electronic temperatures is estimated from modulated SERS spectra, collected at a number of different positions across the sample. For each spectrum, the effective molecular temperature ${T}_{{{{\rm{mol}}}}}$ is calculated via the Stokes/antiStokes intensity ratio (${I}_{{{{\rm{S}}}}}$/${I}_{{{{\rm{aS}}}}}$) of vibrations between 300‒1600 cm^–1^, along with the corresponding electronic temperature ${T}_{e}$ (see Supplementary Note 5). Sensible estimates of average local temperature changes are returned by both approaches, giving $\Delta {\bar{T}}_{{{{\rm{mol}}}}}=\,$85$\pm \,$ K and $\Delta {\bar{T}}_{e}=\,$58$\pm$16 K (Fig. 2d, e). This corresponds to an effective 2D slab thermal conductivity of $\kappa \sim$0.1 Wm^−1^K^−1^ (Supplementary Note 6), which is a thousand-fold smaller than bulk Au, and similar to polymers^53^. However, within a simple model of molecular phonon occupation, temperature increases on this scale cannot also account for the overall decrease of Stokes vibrational and electronic scattering. Such an effect would require depletion of the ground state population due to an optically-induced increase in ${n}_{v}$, typically requiring ultrashort pulsed excitation^54^. For the NO_2_ vibration of NTP, ground state depletion for $\Delta T\sim$ 50 K predicts $|\Delta {I}_{{{{\rm{S}}}}}({{{{\rm{NO}}}}}_{2})|\sim \,$0.5% (Supplementary Note 5.2), far below the observed 30% Stokes decrease.

Rather, it is likely that multiple photothermal effects are present: a simultaneous increase of the effective molecular temperature and a modulation of the s-MLagg plasmonic resonance. The latter affects both the in-coupling of the 785 nm Raman laser and the spectrally-dependent out-coupling of scattered light. If a modulation of the s-MLagg plasmonic resonance occurs by MIR heating of the sample, this could decrease the Stokes scattering strength and the overall SERS intensity. In combination with a thermally-enhanced antiStokes signal, this would subsequently reproduce the observed modulation. Such an effect would also account for the higher temperature change $\Delta {\bar{T}}_{{{{\rm{mol}}}}}$ extracted from molecular vibrations compared to $\Delta {\bar{T}}_{e}$, as the former is sensitive to the relative plasmonic enhancements of ${I}_{{{{\rm{S}}}}}$ and ${I}_{{{{\rm{aS}}}}}$ signals. Indeed, a notably different photothermal response is observed when spectrally tuning the s-MLagg resonance. As it is shifted towards longer wavelengths, both ${I}_{{{{\rm{S}}}}}$ and ${I}_{{{{\rm{aS}}}}}$ signals are now observed to increase with MIR illumination (Supplementary Fig. 3), demonstrating the importance of plasmonic enhancement in determining the photothermal modulation.

### Photothermal response as a probe of nanoscale heat transport

**Fig. 3:**
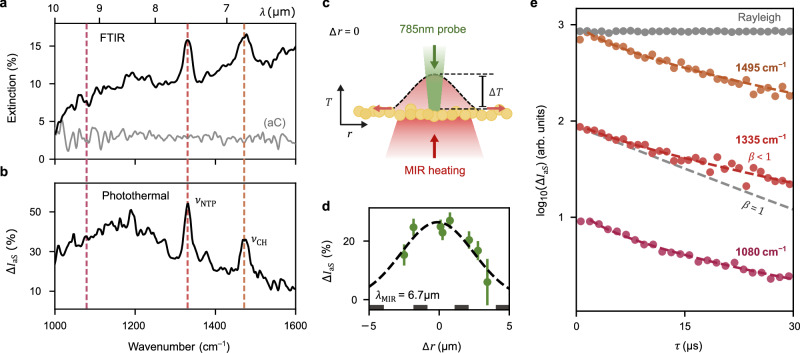
Spectral, spatial and temporal characteristics of photothermal heating. **a** FTIR spectrum of NTP:CB[5] s-MLagg (averaged over 5 positions), compared to bare amorphous C grid (grey). **b** Photothermal modulation signal given by change in integrated antiStokes intensity $\Delta {I}_{{{{\rm{aS}}}}}$ *vs* MIR wavenumber, averaged over 3 positions and normalised by MIR power. Peaks (dashed) from NTP nitro-stretch at ${\nu }_{{{{\rm{NTP}}}}}$ = 1335 cm^‒1^ and CH-stretch of CB[5] at v_(CH) = 1495^‒1^, 1080 cm^‒1^ (dashed) used for off**-**resonant comparison. **c** Spatial overlap of pump and probe in time and space, indicating profile of MIR-induced temperature change $\Delta T$. **d** Integrated antiStokes modulation $\Delta {I}_{{{{\rm{aS}}}}}$ *vs* spatial offset $\Delta r$ between MIR and 785 nm beams at $\tau$=0, with a Gaussian fit. Amorphous C grid indicated in black. **e** Decay of $\Delta {I}_{{{{\rm{aS}}}}}$ after excitation at three different MIR frequencies of 1495 (orange), 1335 (red) and 1080 cm^‒1^ (purple), indicated by corresponding colours in (**a**, **b**). Fits are to the stretched exponential function (see text). Elastic Rayleigh laser scatter *vs* $\tau$ shown as grey points.

The total $\Delta {I}_{{{{\rm{aS}}}}}$ (integrated across all vibrations) is measured by tuning across MIR frequencies ${\nu }_{{{{\rm{MIR}}}}}=\,$1000–1600 cm^–1^ (Fig. 3). The resulting photothermal absorption spectrum shows two peaks around ${\nu }_{{{{\rm{MIR}}}}}=$ 1331 and 1490 cm^‒1^ on a broader background response (Fig. 3b). These peaks are in excellent agreement with the MIR absorption spectrum of the s-MLagg sample, measured by FTIR (Fig. 3a). The two vibrational resonances are assigned to the NTP nitro-stretch mode at ${\nu }_{{{{\rm{NTP}}}}}=$1335 cm^‒1^ (refs. ^55,56^) and CH-stretch mode of CB[5] at ${\nu }_{{{{\rm{CH}}}}}=$ 1495 cm^‒1^ (refs. ^57,58^).

Tuning the MIR frequency, therefore, controls how heat is directed into the nanostructured film. When detuned from vibrational modes, AuNPs dominate the thermal absorption. Resonantly exciting molecular vibrations provides an additional absorption channel, of comparable strength to the non-resonant Au background. The presence of such vibrational signatures within the photothermal spectrum confirms that the modulated signal directly probes the interaction of MIR light with the metal-molecule plasmonic system. This is in contrast with previous experiments^34^ in which similar SERS modulation is rather attributed to an indirect MIR-heating of the substrate material. We also find minimal absorption from the amorphous C grid (Fig. 3a), and the photothermal response of s-MLaggs thus enables a detailed study of how molecular structure and nanogap morphology affect thermal transport in nanoscale systems.

### Thermal decay in s-MLagg

The spatial extent of photothermal modulation follows the MIR beam profile, with a FWHM $d\sim \,$ 8µm when illuminated at MIR wavelength ${\lambda }_{{{{\rm{MIR}}}}}=$ 6.7 µm (Fig. 3c, d). Measurements by the 785 nm probe average the SERS modulation over an area an order of magnitude smaller than the MIR focus. We thus assume a constant $T$ within the probe spot when both beams are perfectly overlapped. At time $\tau$ after MIR illumination, heat decay to the surrounding area gives decaying average temperature $T$. Since $\Delta {I}_{{{{\rm{aS}}}}}$ is directly related to $\Delta T$, thermal decay within the MLagg film is tracked by monitoring the antiStokes signal at increasing 785 nm probe delay $\tau$. This is measured using the CW SERS probe laser with a time-tagged single-photon detector (SPAD) coupled to an FPGA^34,59^, giving sub-µs time resolution after a 400 ns MIR pulse arrives at $\tau$=0. We note that thermal conductivity is related to pump spot size^60^ by $\tau \propto {d}^{2}/\kappa$ so that for fixed $d$, measurements of the decay time track the relative thermal conductivity.

This time-dependent thermal decay (Fig. 3e) is shown for MIR excitation at frequencies ${\nu }_{{{{\rm{MIR}}}}}$ = 1335 cm^‒1^ (resonant with the NO_2_ vibration of NTP), at ${\nu }_{{{{\rm{MIR}}}}}$ = 1495 cm^‒1^ (resonant with the CH vibration of CB[5]), and at ${\nu }_{{{{\rm{MIR}}}}}$ = 1080 cm^‒1^ (where no strong vibrational signal is observed, Fig.3b). The signal is fit to a stretched exponential function^61^ $\Delta {I}_{{{{\rm{aS}}}}}\propto \,\exp [-(\tau /\gamma )^\beta ],$ defined by decay constant $\gamma$. The exponent factor $\beta$ models disorder over a distribution of decays, defined for $0 < \beta \le 1$, with $\beta=1$ corresponding to full order and $\beta < 1$ to more disordered systems. The sensitivity of this technique is compared to direct measurement of the optical reflectance upon heating, well established as a measure of local temperature^62–64^. This reflectivity is recorded from the collected Rayleigh scattering, attenuated to a comparable count rate as the antiStokes signal. No clear modulation or decay is resolved (grey points Fig. 3e), showing how much more sensitive time-resolved thermo-modulation is when combined with the extreme enhancement from light confinement in plasmonic nanogaps.

Comparable decay rates are measured at all MIR excitation frequencies, characterised by a sub-exponential decay with $\beta=$0.7 ± 0.1 and decay constant ${\gamma }_{{{{\rm{NTP}}}}:{{{\rm{CB}}}}[5]}=$13.2 ± 0.8 µs. The disorder implied by $\beta < 1$ is attributed to a distribution in the ratio of NTP:CB[5] molecules within each nanogap. Whilst photothermal modulation is enhanced by the vibrational modes of the system, subsequent decay of $\Delta {I}_{{{{\rm{aS}}}}}$ is independent of the absorption channel. The rate of thermal decay is thus the same when MIR absorption is enhanced by different molecular vibrational modes or indeed when MIR absorption is off-resonant and dominated by Au (Fig. 3e). This indicates a rapid thermalisation within AuNPs (faster than our time resolution), to an equilibrium temperature profile which subsequently decays to cooler neighbouring AuNPs on a much slower timescale.

### Heat transport dependence on nanogap composition

Nanogaps within the s-MLagg correspond to a thermal resistor network, with heat flow efficient within each Au nanoparticle but experiencing a thermal bottleneck across each nanogap when transport swaps from electronic (in the Au) to vibrational (through modes $h\nu < {k}_{B}T$). Convection is estimated to be significantly smaller and is ignored^65^. Dissipation of heat within the MLagg is therefore sensitive to the average thermal conductivity ${\kappa }_{p}$ of each nanogap. Introducing a connecting molecule with a higher ${\kappa }_{p}$ should more rapidly dissipate heat, corresponding to shorter characteristic $\gamma$ in photothermal SERS measurements. The role of molecular heat conduction for thermal transport across plasmonic nanogaps is therefore studied by preparing s-MLagg samples incorporating nanogaps with different thermal properties.

**Fig. 4:**
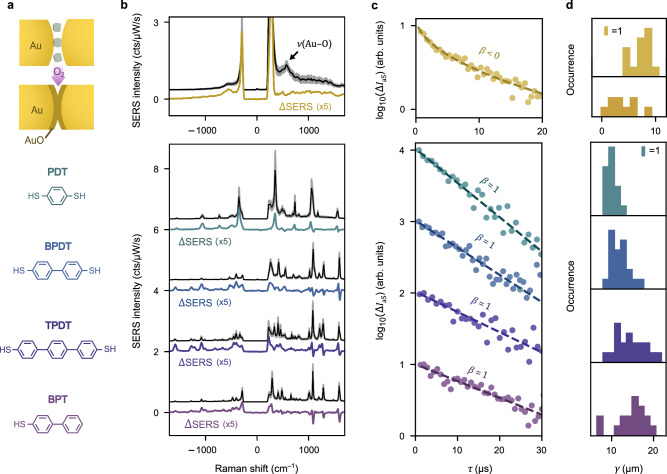
Photothermal SERS modulation with different nanogap composition. **a** Au-oxide (AuO) nanogaps formed by O_2_ plasma exposure. Below, chemical structures of PDT, BPDT, TPDT and BPT molecules used. **b** Normalised SERS intensity (black line) and its variance with position (grey shaded) measured for each sample. Molecular samples are averaged over 10 positions, normalised to 1080 cm^‒1^ peak; AuO are averaged over $7$ positions and normalised to 600 cm^‒1^ peak. $\Delta$SERS shows fractional change of normalised SERS spectra. **c** Temporal decay of $\Delta {I}_{{{{\rm{aS}}}}}$ with exponential best fits. **d** Histograms of extracted decay constants $\gamma$ measured at multiple positions across each sample. All measurements of aromatic molecules use ${\nu }_{{{{\rm{MIR}}}}}=$ 1470 cm^‒1^, in resonance with the infrared-active ring vibration. AuO measurements use ${\nu }_{{{{\rm{MIR}}}}}=$ 890 cm^‒1^ (maximum MIR power) as no strong infrared vibration is present.

We first consider the situation when molecules are replaced by an inorganic oxide. When exposed to O_2_ plasma, all organics are removed from s-MLagg nanogaps, which are instead plugged by a $\sim$2 nm-thick layer of Au-oxide (Au_2_O_3_) (Fig. 4a)^29,30,66^. SERS spectra of the s-MLagg now show only a broad peak at ${\nu }_{{{{\rm{Au}}}}-{{{\rm{O}}}}}\sim$600 cm^‒1^ characteristic of Au–O vibrations (Fig. 4b). MIR illumination gives the same antiStokes increase and Stokes decrease previously observed for s-MLaggs of NTP:CB[5] (Fig.1d). This photothermally-induced antiStokes signal also decays with a stretched exponential response (Fig.4c) which varies slightly at different positions and gives an average decay constant ${\gamma }_{{{{\rm{AuO}}}}}=$7.3 ± 0.5 µs (Fig.4d). A second independently-prepared sample gives ${\gamma }_{{{{\rm{AuO}}}}}=$3.9 ± 0.8 µs, likely due to sensitivity of Au-oxide formation in different local oxidation conditions, and thus different intragap morphologies. Overall, however, heat is transported through these Au-oxide nanogaps >200% faster than through metal-molecule junctions of NTP:CB[5].

Further s-MLagg samples are prepared with one of three di-thiolated aromatic molecules of increasing length: benzene-1,4-dithiol (PDT), biphenyl-4,4’-dithiol (BPDT), or terphenyl-4,4”-dithiol (TPDT) (Fig. 4a). For comparison with BPDT, a fourth sample is prepared with a mono-thiolated molecule biphenyl-1,4’-thiol (BPT), to probe the effects of reducing the overall molecule-metal contact. The homogeneity of nanogap composition is improved by directly aggregating AuNPs with the target molecule (without the presence of CB[5]), confirmed by SERS intensities varying little with spatial position (grey shaded range in Fig. 4b). All photothermal measurements are at MIR frequency ${\nu }_{{{{\rm{MIR}}}}}=$ 1470 cm^‒1^, in resonance with the strongly infrared-active ring vibration possessed by all the molecules. Each shows a clear MIR photothermal modulation characterised by an increased antiStokes intensity, a decreased Stokes intensity, as well as frequency shifts of all vibrational modes as before.

The temporal response of $\Delta {I}_{{{{\rm{aS}}}}}$ now follows a purely exponential decay (Fig. 4c), confirming the improved consistency of thermal properties across these nanogaps. For each molecule, measurement over multiple sample positions (${N}_{{{{\rm{sample}}}}}$ of PDT: 16, BPDT: 20, TPDT: 19, BPT: 17) yields a distribution of $\gamma$ with average values ${\gamma }_{{{{\rm{PDT}}}}}=$9.9 ± 0.3 µs, ${\gamma }_{{{{\rm{BPDT}}}}}=$11.9 ± 0.5 µs, ${\gamma }_{{{{\rm{TPDT}}}}}=$14.6 ± 0.8 µs, and ${\gamma }_{{{{\rm{BPT}}}}}=$15.5 ± 0.6 µs (Fig. 4d). In contrast to previous Au-oxide measurements, average $\gamma$ are consistent between different samples of the same molecule (see Supplementary Note 8). The thermal transport times clearly depend on the length of the bridging molecule and are decreasingly conducting (by 50%) for each ring added. The distribution width of measured $\gamma$ is also found to increase with the number of rings, broadening by a factor of 3 between PDT and TPDT. This increase is not reflected in the spatial variation of SERS intensities, which is comparable for both molecules (Fig. 4b). Instead, it is likely to come from increasing intermolecular disorder, as we will show is important in molecular heat conduction. Reducing the degree of metal-molecule bonding further slows the transport of heat through s-MLaggs, with BPT slowing heat dissipation by 30% in comparison to the structurally analogous molecule BPDT.

### Non-equilibrium molecular dynamics (NEMD)

**Fig. 5:**
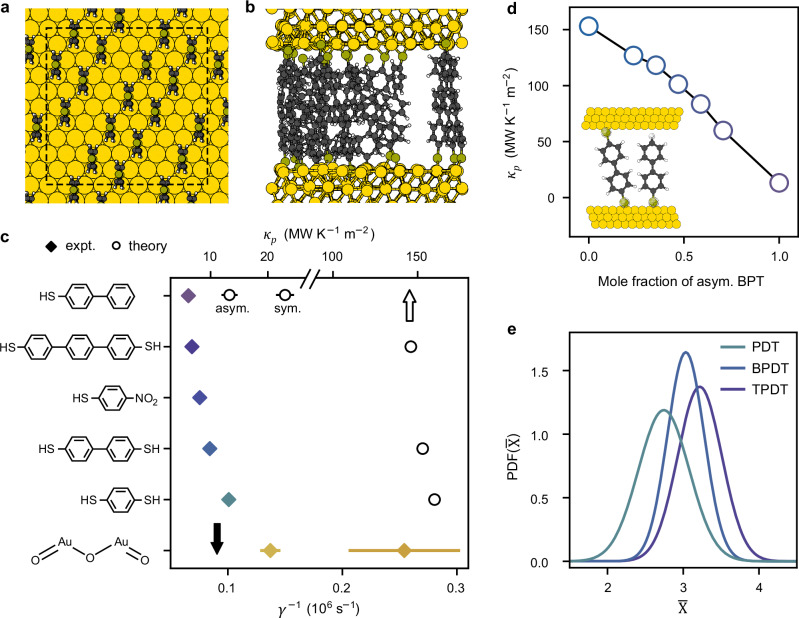
Simulated thermal conductivity through SAMs. **a** Plan view of TPDT SAM junction at the beginning of heat transport simulations. Atoms are: C (grey), H (white), S (olive), and Au (yellow). Simulation cell is black dashed, periodically tiled in $x,y$ directions for clarity. **b** Cross-section of relaxed configuration of TPDT SAM on Au (111) surface. Au atom size reduced for clarity. **c** Experimental decay rates ${\gamma }^{-1}$ (average and uncertainty, bottom axis) along with the thermal conductivity, ${\kappa }_{p}$, obtained from NEMD (top axis). For BPT, two configurations are modelled: all molecules attached to one side (sym), or molecules randomly attached to either Au facet (asym). **d** Thermal conductivity of BPT-BPDT mixture samples as a function of BPT content. (inset) Schematic of mixed BPDT–BPT SAM (1:1 mole fraction), colours as (**a**). **e** Average sulphur coordination number, $\bar{{{{\rm{X}}}}}$, for SAMs with varying number of rings, using a 6 Å cutoff radius to define neighbours.

Having identified the experimental decay constants $\gamma$ of nanogaps formed by thiolated aromatic molecules, we now model heat dissipation across nanogaps containing PDT, BPDT, TPDT or BPT molecules. We adopt a non-equilibrium molecular dynamics (NEMD) simulation to track how thermal vibrations of one metal facet transfer to a colder facet through connecting molecules. To this end, the nanogaps are modelled with two Au electrodes at different temperatures, each composed of 2160 atoms. For each simulation, 17 aromatic molecules are placed on the Au electrodes, with thiol anchor groups attached to the (111) surface at hollow sites (Fig. 5a). Initially, the SAMs are arranged in a $\left(2\times 3\sqrt{2}\right)R23^\circ$ packing configuration, with a molecular footprint (area per molecule) of $38\,{{{\AA }}}^{2}/$ molecule, close to experimental observations for the benzenethiol family^67,68^. During simulations, the molecules can move within the simulation box, and $\pi -\pi$ interactions mediate nanoclustering between neighbouring molecules (Fig. 5b). In all MD simulations of SAM junctions, S atoms can be adsorbed onto either the hot or cold electrodes. A heat flow of 150 meV/ps is used to establish a temperature gradient across the junctions. The thermal conductivity of each junction is then defined as the ratio of heat flow to the temperature gradient per unit area. For each junction, six independent simulations are conducted and used to calculate an average thermal conductivity. More detailed information on the MD simulations and interaction parameters is provided in the Computational Methods section.

The resulting MD-extracted ${\kappa }_{p}$ (Fig. 5c, open points) shows significant differences in heat dissipation between molecules of differing length and number of thiol anchor groups. These trends are qualitatively consistent with our experimental findings (Fig. 5c, solid points): shorter molecules, which exhibit higher thermal conductivity, enable more heat dissipation in the junctions and correspond to faster thermal decay rates (${\gamma }^{-1}$). However, a steeper length dependence is observed in the experimental results and the predicted ${\kappa }_{p}$ underestimates the difference in decay rates for increasing molecular length (PDT to TPDT). Similarly, whilst NEMD simulations correctly predict lower ${\kappa }_{p}$ for mono- *vs* di-thiolated molecules, the BPT and BPDT simulations predict an order of magnitude difference in ${\kappa }_{p}$, significantly larger than the 30% difference measured in $\gamma$ (Fig. 5c).

These discrepancies likely arise from molecular disorder within the measured nanogaps, which is unfeasible to capture through NEMD simulations. In particular, calculations of ${\kappa }_{p}$ assume molecules form a complete thermal connection between planar Au electrodes. However, measured nanogaps possess a $\sim$1 nm surface roughness, previously shown to disrupt the formation of fully-bridged molecular junctions^20,69^. To demonstrate the effect of Au-S contacts on the overall thermal conductance of junctions, we performed NEMD calculations with different ratios of mono-thiolated to di-thiolated molecules (Fig. 5d). Due to a reduced contact resistivity at the interface, increasing the ratio of di-thiolated molecules in the junction significantly improves thermal conductivity. This suggests that heat conduction is contact-dominated and incomplete Au-S bonding provides an explanation of why di- and mono-thiolated molecules have closer thermal conductivities in experiment than in calculations (Fig. 5c). From Fig. 5d, the difference observed when comparing these results for BPT and BPDT can be accounted for if only $\sim$10% of BPDT molecules form fully-bridged Au-S-R-S-Au junctions. This degree of connectivity is comparable to molecular electronics measurements on similar SAMs^45^, which find <5% of molecules carrying current.

In addition to this static disorder, NEMD simulations reveal the dynamic behaviour of SAMs, within which molecular clusters undergo constant reorganisation at the Au surface via the continual making and breaking of Au-S bonds (see Supplementary Movies 1 and 2). To better understand the effect of this dynamic aggregation on thermal conductivity, we analysed our MD trajectories in simulated SAMs with different numbers of alkyl rings. Each molecule is represented by the position of its terminal S atoms. To quantify aggregation, we define a coordination number ${{{\rm{X}}}}$ as the average number of sulphur atoms found within a sphere of radius $R$ = 6 Å centred on each S atom. The mean value $\bar{{{{\rm{X}}}}}$ is computed for each frame of the MD simulation, and a normal distribution is fitted to the resulting data. Simulations show that SAMs of longer molecules (BPDT and TPDT) exhibit a larger value of $\bar{{{{\rm{X}}}}}$ (Fig. 5e), indicating a higher degree of molecular aggregation. Similar trends for other radii (5 Å ≤ $R$ ≤ 9 Å) are also seen (Supplementary Fig. 8).

Previous studies of thiolated aromatic molecules have suggested aggregation within SAMs to have a similar length dependence^67^. Such behaviour could explain why a steeper length-dependence of the thermal decay rate is observed in experiment compared to calculations. Aggregated SAM clusters have fewer degrees of freedom to adjust to surface roughness, and therefore, fewer molecular junctions are expected where both S atoms are connected at the Au interface. On average, molecules of increasing length therefore form fewer fully-bridged Au-S-R-S-Au junctions. Without full thermal contact across the gap, heat transport proceeds through intermolecular coupling to neighbouring molecules attached to the opposite electrode^70^. This process is inherently less efficient, leading to a more pronounced reduction in experimentally measured rates of thermal decay. This result shows that conformational disorder strongly influences the average connectivity of molecular SAMs and requires equal consideration, along with the intrinsic thermal conductivity, in optimising the efficiency of nanoscale heat flow within molecular systems.

In conclusion, we developed a sensitive optical technique that can track thermal transport in situ through molecules embedded inside plasmonic structures, with a few-degree temperature resolution, sub-µm spatial resolution, and sub-µs time resolution. By developing free-standing plasmonic nanoparticle sheet structures, we eliminate substrate conduction and are able to study thermal transport through several hundred nanogaps in series and parallel. We compare self-assembled molecular monolayers of different species and show a consistent trend in thermal conductivity, which is similar to that produced by molecular dynamics simulations. Disagreements are likely explained by the strong role of both static and dynamic molecular disorder. The ability to straightforwardly measure thermal conductivities of molecular layers spanning nanogaps gives both a way to access the intriguing predictions from theory regarding roles of phonon interference or ionic gating, while also providing a route to test technological applications such as improved molecular electronics devices, thermal coatings, and thermal emitters.

## Supplementary information

Supplementary Information

Description of Additional Supplementary Files

Supplementary Movie 1

Supplementary Movie 2

Transparent Peer Review file

## Data Availability

The data generated in this study have been deposited in the Cambridge Open Data database under accession code 10.17863/CAM.129811.
