# Supplementary material for: Thermal transport through molecular monolayers in plasmonic nanogaps
Source: Nat Commun. 2026 May 22;17:6737. doi: 10.1038/s41467-026-73256-0 (PMC13385814; doi:10.1038/s41467-026-73256-0)
Supplement: Supplementary file 1 — Supplementary Information [file 41467_2026_73256_MOESM1_ESM.pdf]

Thermal transport through molecular monolayers in plasmonic nanogaps

Fiona Bell[1], Erfan Norouzi Farahani[2], Yeeun Roh[1], Zhenyao Jiang[1], Sara Sangtarash[2], Zhenyao Jiang[1], Hatef Sadeghi[2*], and Jeremy J Baumberg[1*]

[1] NanoPhotonics Centre, Cavendish Laboratory, Department of Physics, JJ Thompson Avenue, University of Cambridge, Cambridge, CB3 0US, United Kingdom

[2] Quantum Device Modelling Group, School of Engineering, University of Warwick, Coventry, CV4 7AL, United Kingdom

[*]email: hatef.sadeghi@warwick.ac.uk; jjb12@cam.ac.uk

S1: Experimental methods
S2: Near-complete replacement of CB[5] with NTP in NP aggregates
S3: Photothermal measurements of NP aggregates on CaF_2
S4: SERS modulation with different NP size
S5: SERS temperature fitting
S6: Estimation of s-MLagg 2D thermal conductivity
S7: Additional SEM images of s-MLagg
S8: Measurement of additional BPT s-MLagg sample
S9: Non-equilibrium molecular dynamics simulations

Supplementary Methods

Supplementary Note 1. Experimental methods

Sample preparation:
Suspended monolayer aggregates (s-MLaggs) are initially formed by adding 500µL of chloroform ($CHCl_3$) and 150 µL of citrate-capped 60nm AuNPs (BBI Solutions) to an Eppendorf tube. Aggregation is initiated by the addition of CB[5] (100µL of 1mM solution), aided by vigorous shaking for ~1 min. Allowing the mixture to settle, the immiscible $CHCl_3$ and aqueous phases separate, forming an interface where aggregated AuNPs settle. The aqueous phase is washed by the addition and removal of 300µL DI water, repeated a total of three times to sufficiently dilute citrate salts and remove excess surfactants. Careful removal of the aqueous phase concentrates the aggregate to a dense bead, floating on the $CHCl_3$ surface. The droplet is extracted and deposited on a clean amorphous carbon substrate before drying overnight. Once dry, samples are immersed overnight in a 1mM solution of NTP before being rinsed with ethanol and dried with N_2.

For direct aggregation of AuNPs with target molecules, the optimal concentration of molecular solution is highly dependent on structure and Au-affinity of the anchor group. When aggregating using BPT, 150µL of 1mM solution is typically required for optimal MLagg formation. Dithiolated molecules (e.g. BPDT) require 150µL of 0.1mM solution. For samples prepared in this way, using both mono- and dithiolated molecules, spatial variation in measured SERS is reduced by soaking dried s-MLagg samples in 10mM solution for at least 4 hours. After this time, the sample is washed with ethanol and dried using N_2.

FTIR spectroscopy:
Measurement of infrared spectra is performed using a commercial Fourier transform infrared (FTIR) microscope (AIM-9000, Shimadzu). Infrared light from a thermal globar lamp is focused onto the sample by a reflective Cassegrain objective (0.7NA, 15x) and spectra are measured in transmission by a liquid nitrogen cooled mercury cadmium telluride (MCT) detector (spectral resolution $4cm^{-1}$). Measurements represent the average of 20µm square area on the sample, with reference to a clean amorphous carbon grid.

Sample excitation:
Two counter-propagating objectives deliver laser power to the sample plane in a customised commercial microscope (BX51, Olympus). From above, a high-NA darkfield objective (0.9NA 100x, Olympus) focuses visible light to a diffraction limited spot size whilst a reflective objective (0.5NA 40x, Thorlabs) focuses MIR power from below to a focal spot size of $\sim 2\lambda_{\mathrm{MIR}}$. The MIR induced signal is probed by SERS of a 785nm CW laser (MatchBox, Integrated Optics Ltd.), collected from above. The reflected laser signal is removed by two 785nm notch filters (785-20 NNF, Iridian) and directed to a visible spectrometer (i303, Shamrock, 600 lines/mm grating) and EMCCD (Newton, Andor) or to a single-photon avalanche diode (SPAD) for highly sensitive, time-resolved detection (SPD-100-CTD, Micro Photon Devices).

Two pulsed MIR excitation sources are used. Initially we use an 2ps OPO (APE) that generates tuneable MIR pulses at 80MHz repetition. Since the pulse separation is far shorter than any thermal relaxation time, this is a quasi-CW MIR excitation. Secondly, a tuneable quantum cascade laser (QCL) outputs a modulated MIR source across the broad wavelength range from 5.4 - 12.8µm, with 0.1nm linewidth and average

power of 3mW (LaserTune, Block Ltd.). Measurements presented in **Figs.1-2** are taken using the ps MIR, whilst those in **Fig.3** are taken with the modulated QCL source using a pulse duration of 150ns and pulse separation of 0.3µs (repetition rate of 3.3 MHz). Both of these measurement configurations are equivalent to quasi-CW MIR excitation. Time resolved measurements shown in **Fig.3e** and **Fig.4** are taken using the QCL MIR source with pulse duration of 400ns and pulse separation 40µs (repetition rate 250kHz).

For time resolved measurements, single-photon detection is operated in conjunction with a field programmable gate array (FGPA) board (Digilent Arty Z7), configured to record the arrival time of individual photons. During FPGA operation, the timestamps of detected photons and MIR reference signal from the QCL are continuously recorded. From this, the time delay between incidence of a MIR pulse on the sample and detection of a photon in the collection path is individually calculated for all recorded photon timestamps. A time-correlated histogram of photon arrival times is populated mapping the evolution of signal intensity after MIR excitation in a fashion analogous to time-correlated single-photon counting (TCSPC) techniques. A full description of the FPGA design and operation can be found in ref.[1].

Computational methods:

All molecular dynamics simulations were conducted using the LAMMPS MD software[2,3] with Nose-Hoover thermostat and barostat[4,5] and visualisation carried out using OVITO.[6] Our NEMD method has been introduced in a previous study[7] however some simulation details were modified to study the SAM junctions in the current work. Relaxed geometries were created using multiple processes. First, a Hessian-free truncated Newton algorithm was used to minimise the energy of each system. Then, the temperature and pressure of the simulation box were set to the room temperature vacuum condition, using a 1 ns (1000000 MD timestep) simulation in the canonical ensemble, followed by another 1 ns simulation in the isothermal–isobaric ensemble. This equilibrium process is necessary to ensure the same molecule–electrode contact for all molecules with different lengths (1,2, and 3 benzene rings).

Heat transport MD simulations were conducted under the microcanonical ensemble for all samples. The phonon thermal conductance of the junctions was defined as $G_p = J/(T_h - T_c)$, where J is the heat flow and T_h and T_c are the temperatures of the hot and cold electrodes respectively. Heat transport simulations continued for 15.5 ns (tested to have minimal effect on the value of G_p). The first 2.5 ns was excluded to eliminate transient effects. T_h and T_c were computed by averaging the temperatures in hot and cold electrodes respectively, during the heat transport simulation. Each NEMD simulation was performed with six different random seeds for the initial velocity distribution. For each sample, the average of the six independent simulations was presented with a 95% confidence level. Both bonded and non-bonded interaction parameters of the organic molecules were generated using AmberTools24.[8] EAM potential[9] and an Au-S Morse potential[10] were selected to model interactions in the gold leads and the organic-metal contact.

Supplementary Note 2. Near-complete replacement of CB[5] with NTP

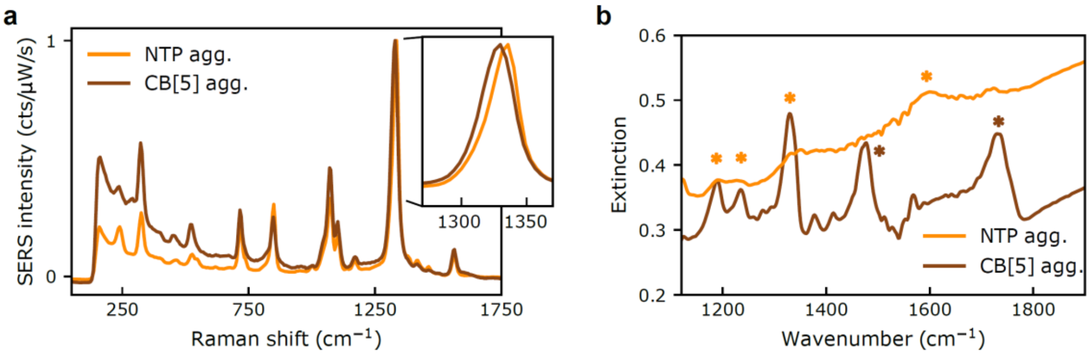

Supplementary Fig. 1 Spectral evidence for near-complete of CB[5] by NTP in s-MLagg samples. Comparison of (**a**) SERS and (**b**) FTIR spectra when comparing two different methods of AuNP aggregation: one directly with NTP molecules (orange) and one in which AuNPs are initially aggregated *via* CB[5], followed by immersion in NTP solution (brown). Inset in (**a**) highlights NO_2 mode of NTP, which exhibits a frequency shift depending on the aggregation method due to sensitivity of particular vibrations on local molecular order. In (**b**), peaks indicated by orange ($*$) are attributed to NTP vibrations, whereas those only in the CB[5] aggregated sample (brown ($*$)) are attributed to CB[5] vibrations.

Supplementary Note 3. Photothermal measurements of NP aggregates on CaF_2

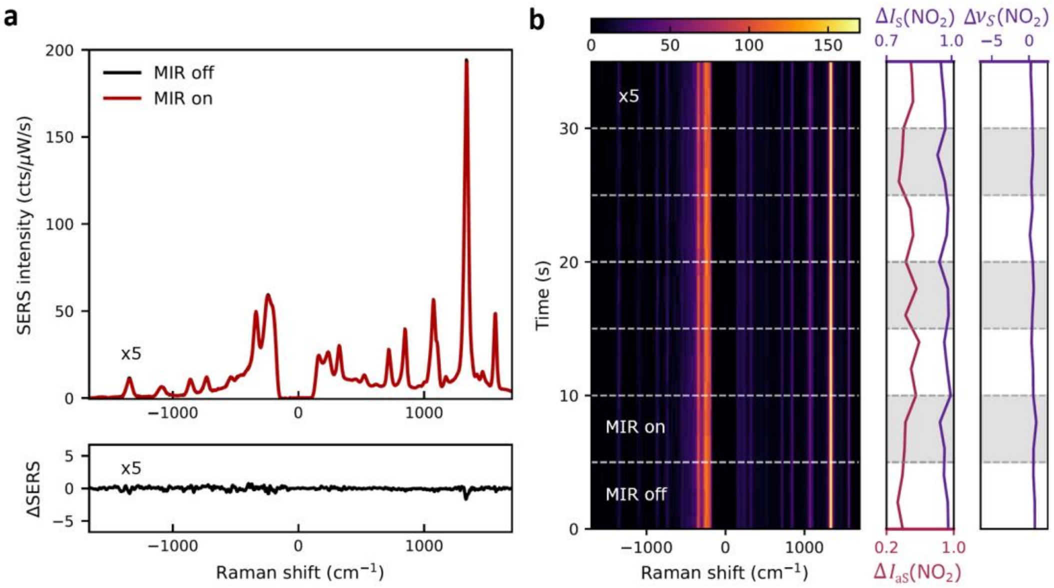

Supplementary Fig. 2 Photothermal modulation of NTP:CB[5] MLagg deposited on CaF_2 substrate. (**a**) SERS spectra under MIR illumination at frequency 1335cm^{-1}, with induced SERS change (ΔSERS) shown on same scale as **Fig.1e** of main text. (**b**) SERS spectra over time, with MIR alternating off/on every 5 spectra. Insets (right) track modulation of Stokes ΔI_S and antiStokes ΔI_{aS} NO_2 peaks. Intensity normalised to initial MIR-off spectrum and traces are indicated on the same scales as used in **Fig.2a** of the main text. Intensities at negative wavenumbers (aS) in (**a,b**) multiplied by x5.

Supplementary Note 4. SERS modulation with different NP size

The effect of s-MLagg plasmonic resonance on SERS photothermal modulation is studied by producing an additional NTP:CB[5] sample, formed by the aggregation of larger 100nm diameter AuNPs. Both 60- and 100nm s-MLaggs are excited using the QCL MIR source, aligned to a frequency of ν_{MIR}=1335cm^{-1}, with pulse duration 150ns and repetition rate 3.3MHz. The change in SERS (ΔSERS) with MIR is measured across several positions, along with the darkfield (DF) scattering response of both samples (**Supplementary Fig. 3**). The latter characterises the plasmonic resonance of the AuNP film at optical frequencies, which determines the spectral enhancement of Raman signals. Sufficient signal-to-noise requires averaging over many sample positions (>15), and spatial variation of the resonance position may broaden the observed response.

The 100nm s-MLagg demonstrates a different photothermal response to the 60nm sample. Whilst both exhibit positive ΔSERS at antiStokes Raman shifts, the intensity of Stokes scattering is observed to decrease for 60nm NPs whereas the 100nm sample demonstrates and increased Stokes signal with MIR illumination. In comparison to the 60nm s-MLagg, the DF scattering spectrum of the 100nm sample shows a red-shift of the plasmonic resonance towards more positive Raman shifts. Enhancement of the incident laser and outcoupled Raman scattering is therefore likely to be different between 60- and 100nm s-MLaggs. Changes to the plasmonic resonance with MIR heating will modify the relative enhancement of in/coupled light, modulating the strength of Stokes and antiStokes signals. This is a reasonable explanation of the different ΔSERS observed for 60- and 100nm s-MLaggs, indicating an additional source of photothermal modulation that does not directly arise from an excited thermal phonon population. Importantly, a similar response is observed for measurements of the 60nm sample here (using 150ns MIR pulses) as with 2ps pulsed MIR excitation shown in **Fig.1e** of the main text, ruling out higher-order effects (e.g. ground state depletion) as a cause of observed Stokes intensity decreases.

A MIR-induced change of the average NP gap size or refractive index would modify the s-MLagg plasmonic resonance, causing a shift or (more likely for such a disordered system) a change in the resonance shape. However, due to the weak DF scattering signal measured in experiments, no consistent MIR-modulation could be observed from s-MLaggs and any changes are likely a very small percentage of the total signal.

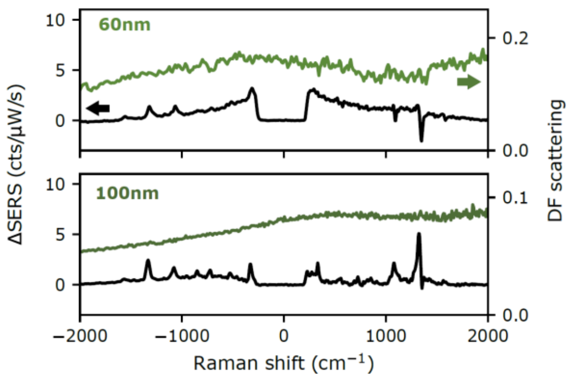

Supplementary Fig. 3 Comparison of average ΔSERS and DF scattering for 60- and 100nm s-MLagg samples (ΔSERS avg. >6, DF scattering avg. > 15 spectra).

Supplementary Section S5: SERS temperature fitting

S5.1 Calibration of SERS spectra

All SERS spectra in this work are spectrally corrected to account for wavelength-dependent transmission efficiency of the collection path. A white-light halogen source is diffusely scattered in the sample plane and the collected spectrum recorded as a reference measurement. Comparison to the known white-light spectrum returns the transmission response function of the given experimental configuration **(Supplementary Fig. 4a**), subsequently used to renormalise collected spectra (**Supplementary Fig. 4b**). Importantly, whilst this procedure addresses any systematic intensity enhancement at different wavelengths, it does not remove additional effects due to plasmonic enhancement which can still modify the relative antiStokes/Stokes SERS intensities. Although it is possible to use dark-field spectra to estimate these plasmonic enhancements, this does not include near-field to far-field conversion efficiencies, and is thus not reliable.[11]

Spectra are further corrected for any systematic error in measured wavelengths (**Supplementary Fig. 4c-e**)**.** The spectral emission from a neon lamp is measured in the sample plane and the position of detected peaks compared to the expected wavelengths from ref.12 (**Supplementary Fig. 4c**)**.** The resulting calibration curve (**Supplementary Fig. 4d**) applies a linear transformation to measured wavelengths, subsequently used in calculation of Raman shifts (**Supplementary Fig. 4e**).

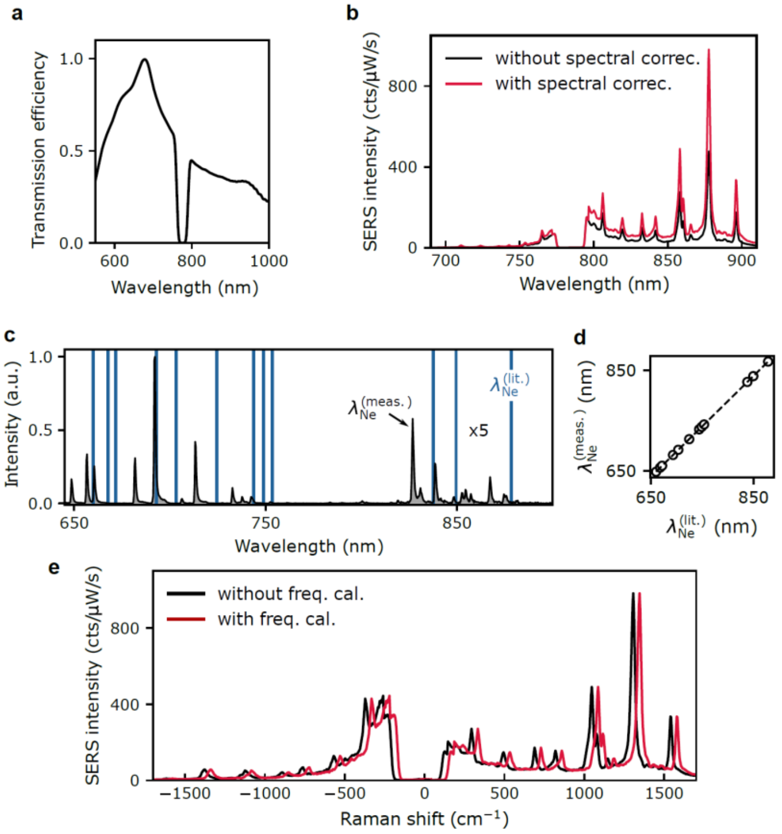

Supplementary Fig. 4 Calibration of SERS (a,b) intensity and (c-e) wavelengths in measured spectra. (**a**) Wavelength-dependent response function of experimental setup described within this work, used to renormalise intensity of collected spectra shown in (**b**). (**c**) Measured spectrum of neon lamp (black) used in wavelength calibration, compared to expected peak positions (blue). (**d**) Linear calibration curve comparing measured neon peak wavelengths $\lambda_{\mathrm{Ne}}^{(\mathrm{meas.})}$ and literature wavelengths $\lambda_{\mathrm{Ne}}^{(\mathrm{lit.})}$, taken from ref.12.

S5.2 Measurement of molecular temperature

Peaks within measured SERS spectra of NTP:CB[5] s-MLaggs are fit by representing each vibrational mode as a Lorentzian function superimposed on a linear background (**Supplementary Fig. 5**). Individual fits are parameterised by freely varying values corresponding to the peak height, width, and central frequency as well as a background slope gradient and constant offset. For each vibration, the total peak area is extracted at both Stokes and antiStokes Raman shifts and subsequently used to calculate the corresponding temperature of each molecular mode, with and without the presence of MIR. The vibrational mode present at ~800cm^{-1} is not included as the highly asymmetric lineshape did not return consistent fits and introduced a large uncertainty into calculated temperatures.

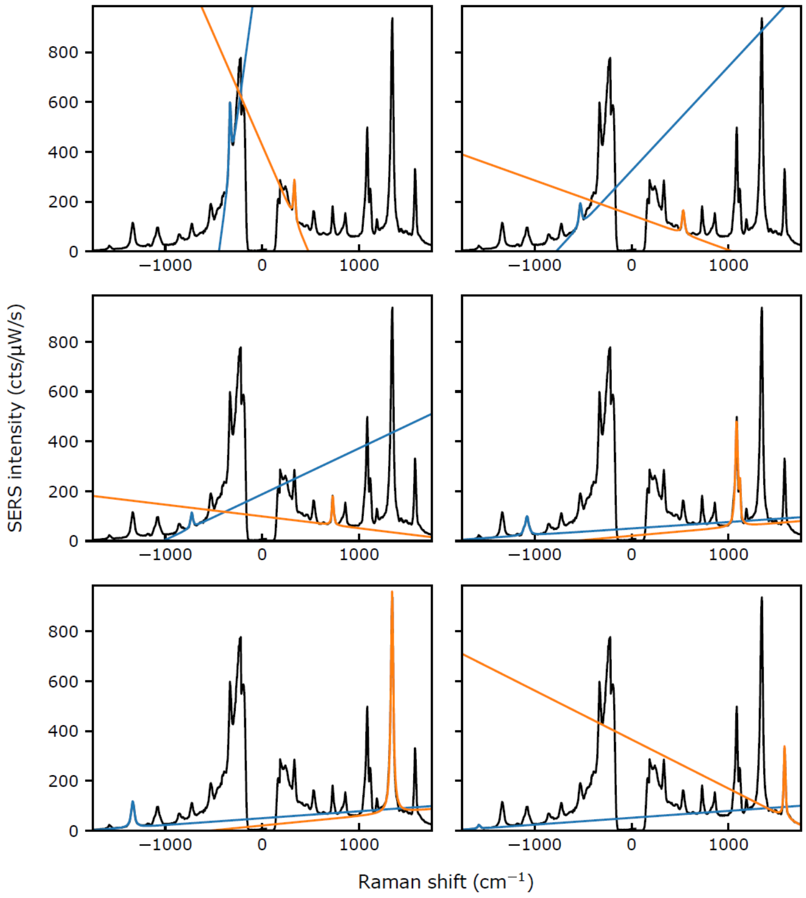

Supplementary Fig. 5 Example results of peak fitting procedure used in the estimation of molecular temperatures. A single Lorentzian fit is applied for all peaks, barring the 1080cm^{-1} vibration for which a fit comprising of two

Lorentzian modes is used. Intensity at negative Raman shifts is here multiplied by x5. Spectra here are taken with MIR off.

For a Stokes intensity (peak area) I_{S} and antiStokes intensity I_{aS}, the molecular temperature T_{mol} is given by[13]

$$T_{\mathrm{mol}} = \frac{\hbar\omega_\nu}{k_B}\left[\ln\left(\frac{\omega_{\mathrm{aS}}^4\, I_{\mathrm{S}}}{\omega_{\mathrm{S}}^4\, I_{\mathrm{aS}}}\right)\right]^{-1}, \tag{1}$$

where ω_ν is the frequency of the vibration, related to the frequency of Stokes/antiStokes signals $\omega_{\mathrm{aS/S}}$ via the laser frequency ω_l as $\omega_{\mathrm{aS/S}} = \omega_l \pm \omega_\nu$, $\hbar$ is the reduced Plancks constant and k_B is the Boltzmann constant. To reduce uncertainty, ω_ν of each mode is determined by the Stokes frequency (after wavelength calibration) which has greater signal-to-noise compared to the antiStokes peak. Example calculations of T_{mol} for all vibrational modes are shown in Supplementary **Table 1** for SERS collected at a single sample position. Equivalent fitting procedures were used for SERS collected at a further three sample positions to calculate average values in **Fig.2d** of the main text. Due to uncertainty in the origin of the Stokes decrease upon MIR illumination (see Section S4), calculations of T_{mol} for both MIR on and off use the MIR-off I_{S} value. For the values in Supplementary **Table 1**, the difference in resulting T_{mol}(MIR on) is <4% compared to using values of I_{S}(MIR on) in calculations.

It is important to note that the temperatures calculated in Supplementary **Table 1** are significantly above room temperature (298 K), even without MIR heating. This artefact likely originates from the plasmonic resonance of the s-MLagg, which causes SERS at different wavelengths to experience a different enhancement factor. Practically, this artificially shifts the system to a local effective temperature, out of equilibrium with ambient conditions. It is therefore only meaningful to consider the local temperature change ΔT induced by MIR heating, which is quoted for both molecular and electronic temperatures in the main text.

The depletion of the ground state due to MIR-induced heating can be estimated, assuming an excited state phonon population n_ν described by a Bose-Einstein distribution defined in **Supplementary Equation 2**. For low temperatures, depletion of ground-state population, given by $(1 - n_\nu)$, can be well approximated as $\propto 1 - \exp(-\hbar\omega/k_B T)$. Using the temperatures calculated for the nitro-stretch mode of NTP (1335 cm^{-1}) in Supplementary **Table 1** shows that the depletion of the ground state only increases by $\sim$ 0.5% with the MIR and therefore cannot account for the 30% change in Stokes intensity seen in **Figs.1,2** of the main text.

Supplementary Table 1 Values extracted from fits in **Supplementary Fig. 5** for each vibrational mode present in the SERS spectrum of NTP:CB[5], indicating Stokes/antiStokes intensity and molecular temperature calculated with and without the presence of MIR.

ν (cm^{-1})	ω_S ($10^{15}\cdot$ rads^{-1})	ω_{aS} ($10^{15}\cdot$ rads^{-1})	I_S (a.u.)		I_{aS} (a.u.)		T_{mol} (K)	
			MIR off	MIR on	MIR off	MIR on	MIR off	MIR on
1575cm^{-1}	2.10	2.67	305	291	1.03	3.03	380 ± 12	447 ± 10
1335cm^{-1}	2.15	2.65	936	891	10.7	21.3	398 ± 4	456 ± 5
1079cm^{-1}	2.19	2.60	417	425	7.86	15.4	362 ± 8	436 ± 11
727cm^{-1}	2.26	2.54	117	114	8.34	12.4	375 ± 16	438 ± 18
529cm^{-1}	2.30	2.50	87.6	91.3	13.3	18.2	326 ± 27	399 ± 40
331cm^{-1}	2.34	2.46	168	161	45.4	57.2	339 ± 13	372 ± 22

S5.3 Measurement of electronic temperature

At low Raman shifts, the electronic Raman scattering (ERS) in SERS spectra follows the thermal phonon population of gold, well described by a Bose-Einstein distribution with metallic temperature T_e.[14] The antiStokes ERS intensity $I_{\mathrm{aS}}^{\mathrm{ERS}}$ is therefore initially fit as

$$I_{\mathrm{aS}}^{\mathrm{ERS}} \propto \left[\exp\left(\frac{\hbar\omega_{\mathrm{ERS}}}{k_B T_e}\right) - 1\right]^{-1}, \tag{2}$$

for ERS frequencies ω_{ERS} corresponding to Raman shifts < 600cm^{-1} (**Supplementary Fig. 6**). Visible laser excitation (wavelength 785nm) produces an additional population of 'hot' electrons, out of equilibrium with the metal lattice. However, this signal is centred around the laser frequency ($\omega_l \gg \omega_{\mathrm{ERS}}$) and appears as a constant background at higher Raman shifts in measured SERS spectra. Using T_e extracted from the low wavenumber fit as an initial value, the antiStokes ERS scattering is subsequently fit with the addition of a constant term in **Supplementary Equation (2).**

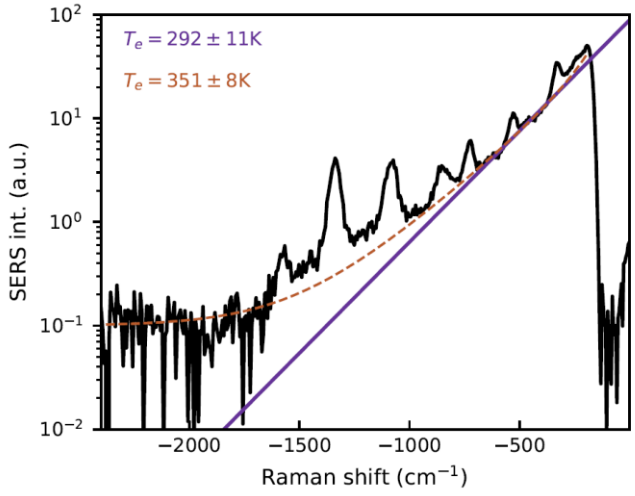

Supplementary Fig. 6 Example fit of SERS background in SERS spectra showing initial Bose-Einstein fit for Raman shifts <600cm^{-1} (purple) and full fit considering background contribution from hot electrons (red). Spectrum taken with MIR off.

Supplementary Note 6: Estimation of s-MLagg 2D thermal conductivity

Illumination of the s-MLagg with MIR laser power P_{MIR}, and focal radius w, induces a maximum central temperature rise ΔT determined by the effective thermal conductivity κ_{eff}. For an equivalent thin 2D-film, this can be estimated as[15]

$$\kappa_{\mathrm{eff}} \sim 2(2\pi)^{-3/2}\alpha P_{\mathrm{MIR}}/(w.\Delta T), \tag{3}$$

with inclusion of a term α representing the intensity fraction absorbed in the film.

Assuming a diffraction limited Gaussian spot, the focal radius is taken as $w \sim \lambda_{\mathrm{MIR}}/2 \sim$ 4µm and, with a power density of 50µWµm^{-2} (see main text), this gives $P_{\mathrm{MIR}} \sim$ 2mW. Using an estimate of α = 0.1 (**Fig.3a** main text) and a value of $\Delta T \sim$ 100K (**Fig.2d** main text) gives

$$\kappa_{\mathrm{eff}} \sim \ 2(2\pi)^{-\frac{3}{2}} \cdot \ 0.1 \cdot 2 \times \frac{10^{-3}}{4\times10^{-6}\cdot 100} \sim 0.06 \ \sim 0.1 \ \mathrm{Wm^{-1}K^{-1}}.$$

Supplementary Note 7: Additional SEM images of s-MLaggs

NTP:CB[5]

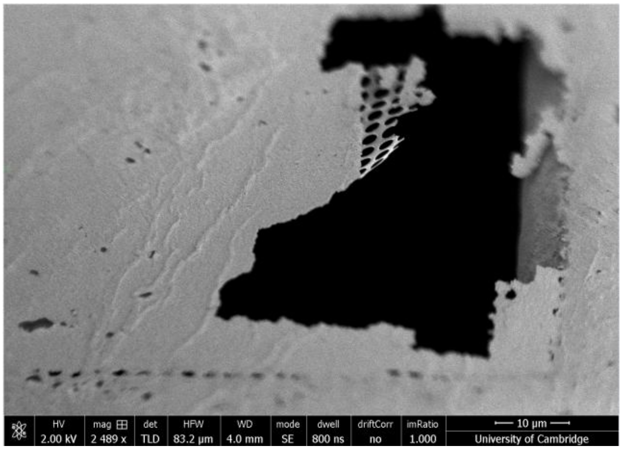

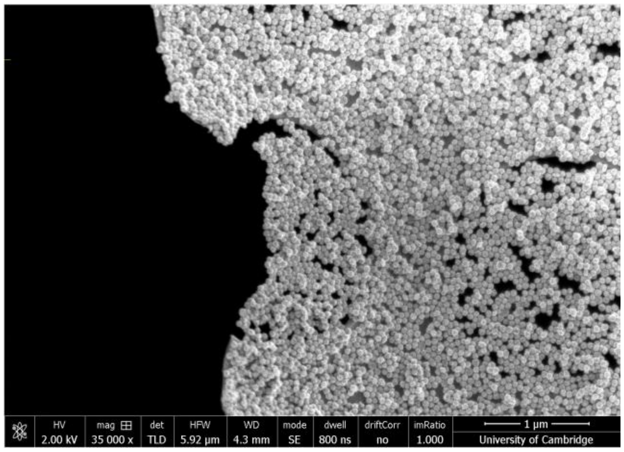

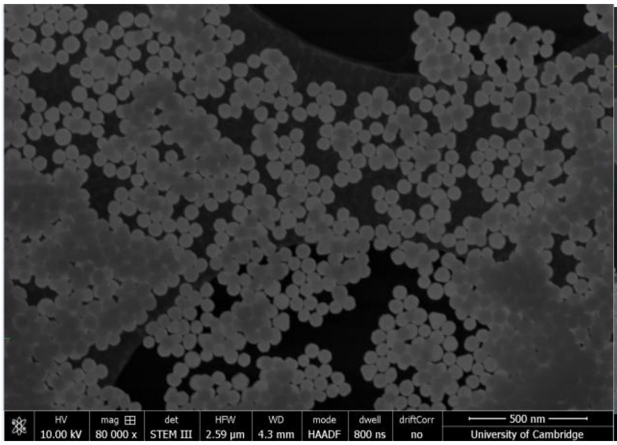

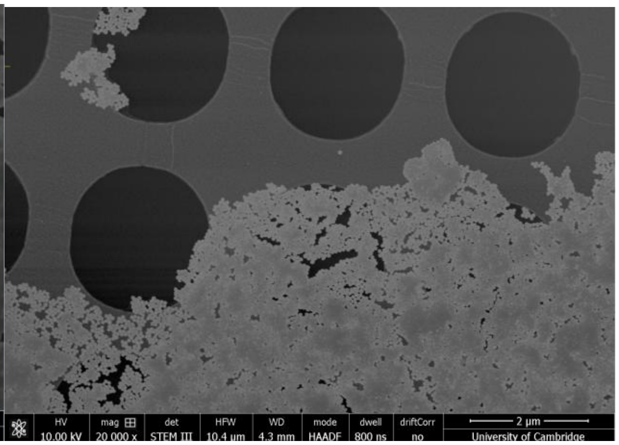

PDT

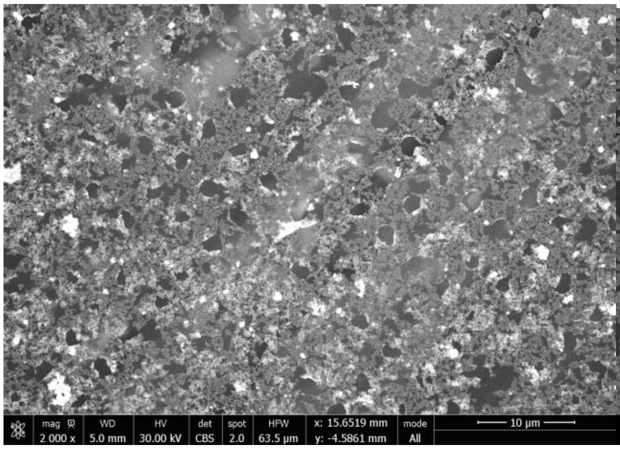

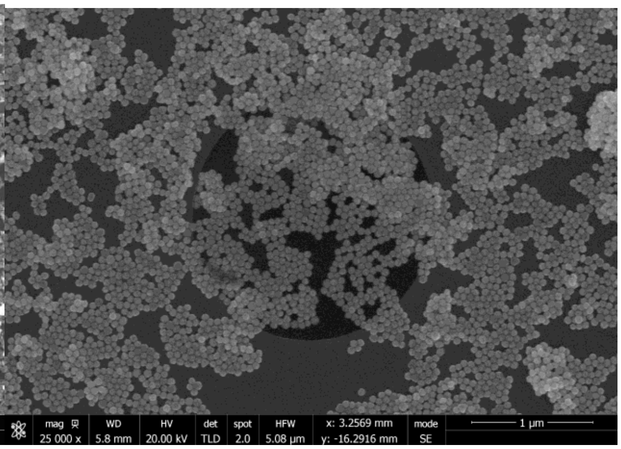

BPDT

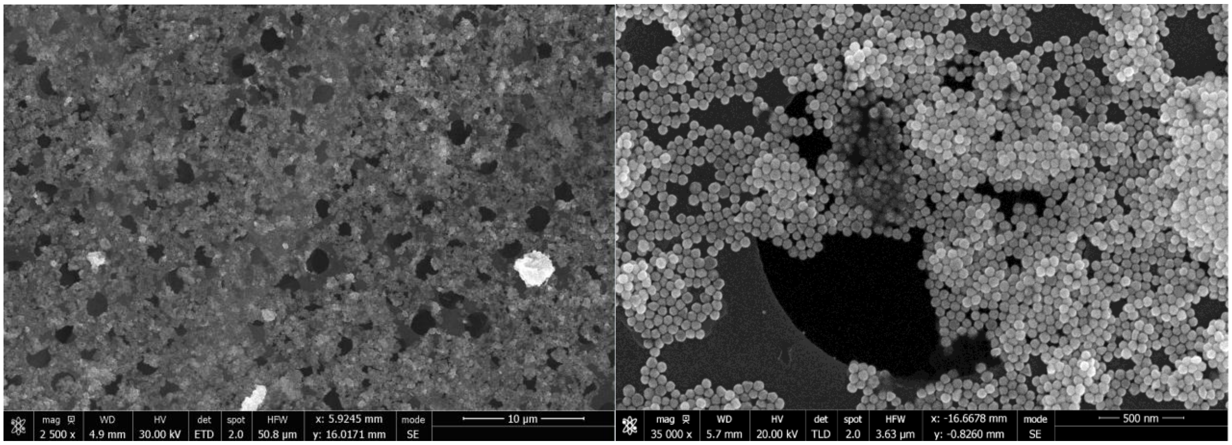

mag WD HV det spot HFW x: 5.9245 mm mode 10 μm
2 500 x 4.9 mm 30.00 kV ETD 2.0 50.8 μm y: 16.0171 mm SE
mag WD HV det spot HFW x: -16.6678 mm mode 500 nm
35 000 x 5.7 mm 20.00 kV TLD 2.0 3.63 μm y: -0.8260 mm SE

TPDT

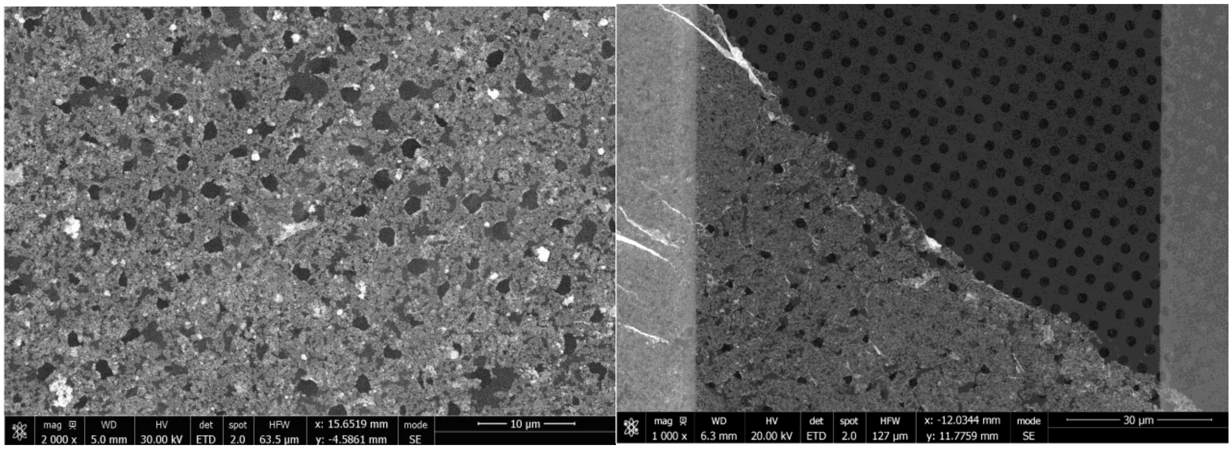

mag WD HV det spot HFW x: 15.6519 mm mode 10 μm
2 000 x 5.0 mm 30.00 kV ETD 2.0 63.5 μm y: -4.5861 mm SE
mag WD HV det spot HFW x: -12.0344 mm mode 30 μm
1 000 x 6.3 mm 20.00 kV ETD 2.0 127 μm y: 11.7759 mm SE

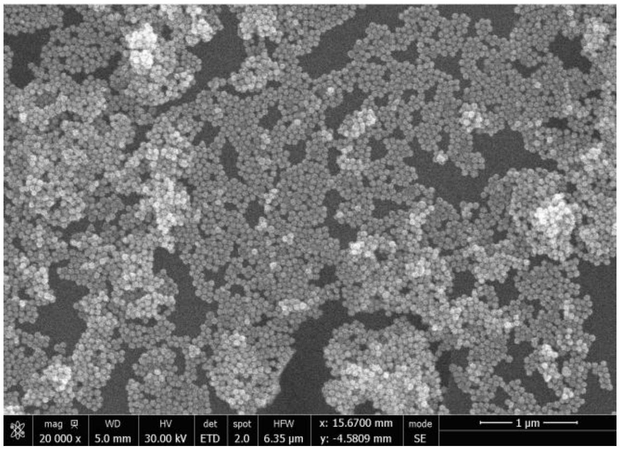

mag WD HV det spot HFW x: 15.6700 mm mode 1 μm
20 000 x 5.0 mm 30.00 kV ETD 2.0 6.35 μm y: -4.5809 mm SE

Supplementary Note 8: Measurement of additional BPT s-MLagg sample

The consistency of γ across different samples of the same molecule is determined by producing an additional BPT s-MLagg. The photothermal decay signal is then measured as described in the main text at a series of sample positions. An example decay is shown in Supplementary **Fig. 7**, fit with a single exponential decay function. Measurements are taken at 5 different positions and resulting fits yield γ = [13.5, 17.1, 18.7, 17.8, 16.8] with an average of γ_{BPT} = 16.8 ± 0.8μs, within error of the previous value γ_{BPT} = 15.5 ± 0.6μs.

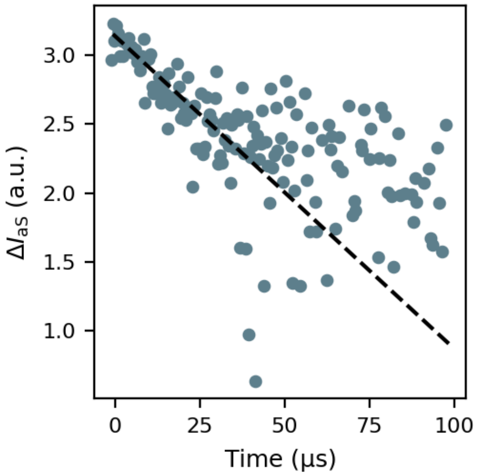

Supplementary Fig. 7 Example photothermal decay of antiStokes signal ΔI_{aS} from additional s-MLagg sample prepared with BPT molecules, exponential fit shown by dashed line. MIR is set to wavenumber ν_{MIR} = 1493cm^{-1}.

Supplementary Note 9: Non-equilibrium molecular dynamics

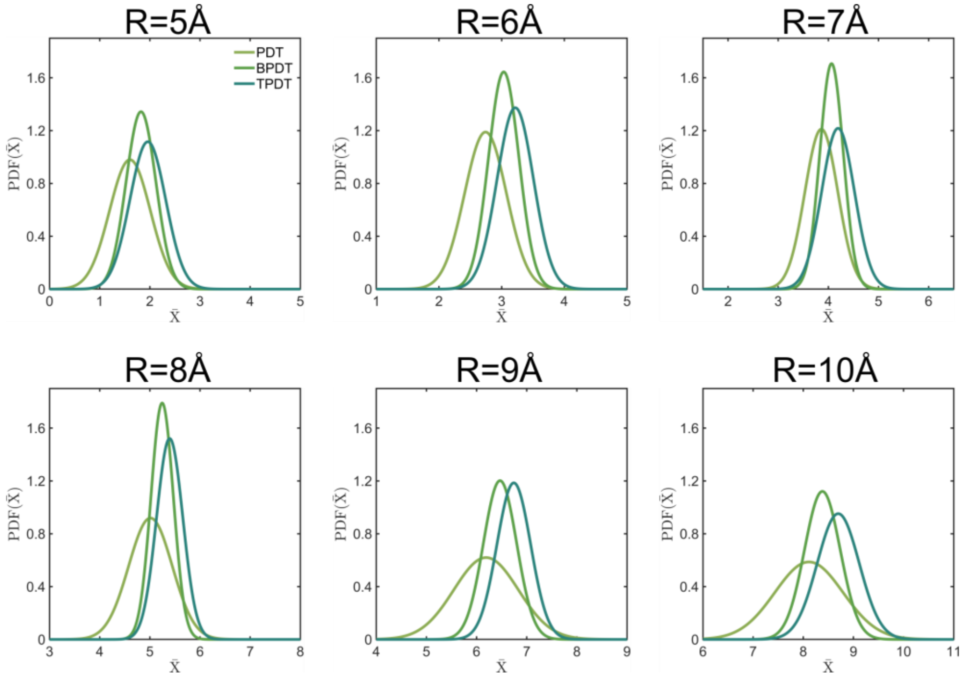

Supplementary Fig. 8. Average sulfur coordination number, X̅, for PDT, BPDT, and TPDT SAMs, calculated using cutoff radii in the range of 5Å ≤ R ≤ 9Å.

Supplementary References

(1) Jakob, L. A.; Deacon, W. M.; Hicks, O.; Manyakin, I.; Ojambati, O. S.; Traxler, M.; Baumberg, J. J. Single Photon Multiclock Lock-in Detection by Picosecond Timestamping. *Optica* **2021**, *8* (12), 1646–1653. https://doi.org/10.1364/OPTICA.441487.

(2) Plimpton, S. Fast Parallel Algorithms for Short-Range Molecular Dynamics. *Journal of Computational Physics* **1995**, *117* (1), 1–19. https://doi.org/10.1006/jcph.1995.1039.

(3) Thompson, A. P.; Aktulga, H. M.; Berger, R.; Bolintineanu, D. S.; Brown, W. M.; Crozier, P. S.; in 't Veld, P. J.; Kohlmeyer, A.; Moore, S. G.; Nguyen, T. D.; Shan, R.; Stevens, M. J.; Tranchida, J.; Trott, C.; Plimpton, S. J. LAMMPS - a Flexible Simulation Tool for Particle-Based Materials Modeling at the Atomic, Meso, and Continuum Scales. *Computer Physics Communications* **2022**, *271*, 108171. https://doi.org/10.1016/j.cpc.2021.108171.

(4) Nosé, S. A Unified Formulation of the Constant Temperature Molecular Dynamics Methods. *The Journal of Chemical Physics* **1984**, *81* (1), 511–519. https://doi.org/10.1063/1.447334.

(5) Hoover, W. G. Canonical Dynamics: Equilibrium Phase-Space Distributions. *Phys. Rev. A* **1985**, *31* (3), 1695–1697. https://doi.org/10.1103/PhysRevA.31.1695.

(6) Stukowski, A. Visualization and Analysis of Atomistic Simulation Data with OVITO–the Open Visualization Tool. *Modelling and Simulation in Materials Science and Engineering* **2009**, *18* (1), 015012. https://doi.org/10.1088/0965-0393/18/1/015012.

(7) Zheng, L.; Norouzi Farahani, E.; Daaoub, A. H. S.; Sangtarash, S.; Sadeghi, H. Rules of Connectivity-Dependent Phonon Interference in Molecular Junctions. *Nano Lett.* **2025**, *25* (16), 6524–6529. https://doi.org/10.1021/acs.nanolett.5c00225.
(8) Case, D. A.; Aktulga, H. M.; Belfon, K.; Cerutti, D. S.; Cisneros, G. A.; Cruzeiro, V. W. D.; Forouzesh, N.; Giese, T. J.; Götz, A. W.; Gohlke, H.; Izadi, S.; Kasavajhala, K.; Kaymak, M. C.; King, E.; Kurtzman, T.; Lee, T.-S.; Li, P.; Liu, J.; Luchko, T.; Luo, R.; Manathunga, M.; Machado, M. R.; Nguyen, H. M.; O'Hearn, K. A.; Onufriev, A. V.; Pan, F.; Pantano, S.; Qi, R.; Rahnamoun, A.; Risheh, A.; Schott-Verdugo, S.; Shajan, A.; Swails, J.; Wang, J.; Wei, H.; Wu, X.; Wu, Y.; Zhang, S.; Zhao, S.; Zhu, Q.; Cheatham, T. E. I.; Roe, D. R.; Roitberg, A.; Simmerling, C.; York, D. M.; Nagan, M. C.; Merz, K. M. Jr. AmberTools. *J. Chem. Inf. Model.* **2023**, *63* (20), 6183–6191. https://doi.org/10.1021/acs.jcim.3c01153.
(9) Grochola, G.; Russo, S. P.; Snook, I. K. On Fitting a Gold Embedded Atom Method Potential Using the Force Matching Method. *The Journal of Chemical Physics* **2005**, *123* (20), 204719. https://doi.org/10.1063/1.2124667.
(10) Mahaffy, R.; Bhatia, R.; Garrison, B. J. Diffusion of a Butanethiolate Molecule on a Au{111} Surface. *J. Phys. Chem. B* **1997**, *101* (5), 771–773. https://doi.org/10.1021/jp962281w.
(11) Hu, S.; Elliott, E.; Sánchez-Iglesias, A.; Huang, J.; Guo, C.; Hou, Y.; Kamp, M.; Goerlitzer, E. S. A.; Bedingfield, K.; de Nijs, B.; Peng, J.; Demetriadou, A.; Liz-Marzán, L. M.; Baumberg, J. J. Full Control of Plasmonic Nanocavities Using Gold Decahedra-on-Mirror Constructs with Monodisperse Facets. *Advanced Science* **2023**, *10* (11), 2207178. https://doi.org/10.1002/advs.202207178.
(12) Kramida, A.; Ralchenko, Yu.; Reader, J.; NIST ASD Team (2024). NIST Atomic Spectra Database (Ver. 5.12). *National Institute of Standards and Technology*, 2024. https://physics.nist.gov/asd.
(13) Le Ru, E. C.; Etchegoin, P. G. *Principles of Surface-Enhanced Raman Spectroscopy*; Elsevier: Amsterdam, 2009.
(14) Boehmke Amoruso, A.; Boto, R. A.; Elliot, E.; de Nijs, B.; Esteban, R.; Földes, T.; Aguilar-Galindo, F.; Rosta, E.; Aizpurua, J.; Baumberg, J. J. Uncovering Low-Frequency Vibrations in Surface-Enhanced Raman of Organic Molecules. *Nature Communications* **2024**, *15* (1), 1–10. https://doi.org/10.1038/s41467-024-50823-x.
(15) Cormier, S.; Salmon, A. R.; Kos, D.; Baumberg, J. J. A Light-Switchable Liquid Metamaterial Mirror. *Advanced Optical Materials* **2020**, *8* (17), 2000396. https://doi.org/10.1002/adom.202000396.
