# Supplementary material for: Thermal transport through molecular monolayers in plasmonic nanogaps
Source: Nat Commun. 2026 May 22;17:6737. doi: 10.1038/s41467-026-73256-0 (PMC13385814; doi:10.1038/s41467-026-73256-0)
Supplement: Supplementary file 2 — Description of Additional Supplementary Files [file 41467_2026_73256_MOESM2_ESM.pdf]

Description of Additional Supplementary Files

File Name: Supplementary Movie 1
Description: NEMD simulation of molecules of different length (BDT,TPDT) to show the dynamics at room temperature, in side and plan views, with unit cell of simulation as marked.

File Name: Supplementary Movie 2
Description: NEMD simulation of molecules with either a thiol at one end (asymm), or at both ends (symm) using BDT,TPDT to compare the dynamics at room temperature, in side and plan views, with unit cell of simulation as marked.
