# Supplementary material for: Thermal transport through molecular monolayers in plasmonic nanogaps
Source: Nat Commun. 2026 May 22;17:6737. doi: 10.1038/s41467-026-73256-0 (PMC13385814; doi:10.1038/s41467-026-73256-0)
Supplement: Supplementary file 5 — Transparent Peer Review file [file 41467_2026_73256_MOESM5_ESM.pdf]

nature portfolio

Peer Review File

Thermal transport through molecular monolayers in plasmonic nanogaps

Corresponding Author: Professor Jeremy Baumberg

This file contains all reviewer reports in order by version, followed by all author rebuttals in order by version.

Version 0:

Reviewer comments:

Reviewer #1

(Remarks to the Author)
The authors describe their experiments on thermal transport in 2D arrays of Au nanoparticles linked by organic molecules. I do not recommend publication. The authors are misinterpreting their experiments. The time-scale they observe for the temperature decay (on the order of 10 microseconds) is many orders of magnitude too long to be physically reasonable for the mechanism that they discuss. What I suspect is happening is that the 2D array of Au nanoparticles that is suspended over the 2 micron openings in the carbon grid is significantly heated by the 400 ns MIR laser pulse and then cools by lateral heat conduction and heat transfer into the carbon grid on a time scale governed by heat diffusion in the Au nanoparticle array on a length scale of 1 micron. Perhaps heat diffusion on the lateral scale of the MIR heating beam (4 micron 1/e^2 radius) is also involved. The authors could distinguish this mechanism from their proposed mechanism by changing the size of the holes in the carbon grid and changing the focus diameter of the MIR laser pulse.

The authors do not clearly explain what they think is happening to the temperature field in the samples. The authors are measuring a decay of the temperature of the molecules. I initially thought that the MIR beam is heating the molecules more than the Au nanoparticles and the authors were proposing that the molecules then cooled slowly into the adjacent Au, but the authors also state that changing the MIR wavelength gives the same result. That seems inconsistent with the discussion surrounding the cooling of the molecules into the Au nanoparticles. In any case, the cooling of molecules would be extremely fast, on a time scale <100 picoseconds, four orders of magnitude shorter than the time resolution of the measurement.

Reviewer #2

(Remarks to the Author)
The authors characterized thermal conduction in a molecular junction by using the SERS technique, showing direct measurement of molecular heat transport. The optical experiments were conducted under precise control, and the analysis results are interesting. The researchers have tried to extract pure thermal transport from SERS utilizing molecular monolayers with plasmonic nanogaps, where many physical phenomena are entangled. While time-resolved photothermal measurements with nanoscale precision in this manuscript would be of great interests, several major points need to be clarified and revised for the publication in Nature Communications.

1. It would be helpful to explain the main idea of how utilizing 2D NP sheets called MLagg can enable the measurement of thermal transport and what the relevant parameters are for the characterization of the nanostructures with more detailed definition of MLagg.

2. Please clarify the contradiction between the statement in line 65 and the Supplementary Section S2 (near-complete vs. partial replacement). Which one is correct? How do you conclude that these Raman and FT-IR show near-complete replacement of CB[5] into NTP throughout the sample?

2. Please add references for the wavenumbers in lines 157, 159, and 160.

3. Since the authors characterized thermal energy transport based on the SERS technique, the distance between adjacent gold nanoparticles can be critical. Please provide the spectroscopic/microscopic data that can provide statistical information

on the distance between gold nanoparticles, depending on the type of junctions.

4. Figure 2 discusses the frequency shift data, which is not explained in relation to the nanogaps or distance between the gold nanoparticles. It would be helpful to provide explanations about those effects on thermal energy transport.

5. The NEMD simulation section can be improved by correlating with experimental results. For example, it is not clear how authors know that the surface roughness of the gold contact leads to fewer fully-bridged Au-S-R-S-Au junction. Some explanations in the paragraph following this sentence are speculative, not substantiated by experimental results or prior results with references.

6. In the conclusion section, it is not clear whether authors can claim that substrate conduction is eliminated as the spot size of MIR may be larger than the hole size of a-carbon grid, which is not discussed in detail. Also, it is not clear whether the technique can be claimed as an ultrasensitive photothermal SERS since the developed technique is for the characterization of sensitive heat conduction for the known molecule.

7. Please improve writing and polish grammar for the overall manuscript, including:
- There should be space between numbers and units.
- Please separate materials and methods into another section to improve readability.

Reviewer #3

(Remarks to the Author)
I co-reviewed this manuscript with one of the reviewers who provided the listed reports. This is part of the Nature Communications initiative to facilitate training in peer review and to provide appropriate recognition for Early Career Researchers who co-review manuscripts.

Reviewer #4

(Remarks to the Author)
This manuscript presents an innovative experimental approach for probing thermal transport through molecular monolayers embedded in plasmonic nanocavities. By combining mid-infrared photothermal excitation with surface-enhanced Raman scattering thermometry in suspended sheets of molecule-connected gold nanoparticles, the authors demonstrate a novel ensemble-based method capable of tracking local temperature dynamics with microsecond temporal resolution. The experimental results are carefully executed and are further strengthened by non-equilibrium molecular dynamics simulations, which capture key trends in molecular-length- and connectivity-dependent heat transport. Overall, the work represents a significant methodological advance with clear relevance to nanoscale thermal management and molecular thermoelectrics.

Despite these strengths, several aspects of the physical interpretation and experimental analysis require further clarification before the conclusions can be fully supported. In particular, the attribution of the observed SERS modulation predominantly to thermal effects would benefit from a more rigorous discussion of alternative mechanisms and a clearer cause-and-effect framework linking temperature rise, plasmon resonance modulation, and vibrational responses. In addition, uncertainties in temperature extraction, potential contributions from non-thermal processes, and aspects of sample heterogeneity and data interpretation require further justification or experimental support. Addressing these points would substantially strengthen the robustness and generality of the reported conclusions.
Individual comments are listed as follows:
1. The authors report an increase in anti-Stokes SERS intensity upon MIR illumination (e.g., Fig. 1d and 2a) and attribute this primarily to an increased phonon population due to thermal excitation. However, similar increases in anti-Stokes emission have been observed in plasmonic systems arising from non-thermal mechanisms, such as molecular frequency up-conversion (e.g., Science 374, 1268–1271 (2021)), vibrational pumping, picocavity formation, inelastic tunnelling, and molecular optomechanical effects. The authors should clarify how these alternative mechanisms are excluded in the present experiments and justify why the observed SERS modulation can be attributed predominantly to thermal effects.

2. The discussion of temperature-induced changes in Stokes vibrational and electronic scattering (In SI and Fig. 1 and 2) reads largely as a list of observations. While the authors dismiss ground-state depletion associated with increased vibrational population and instead emphasize plasmonic modulation, the physical relationship between temperature rise, plasmon resonance modulation, and the observed changes in SERS intensity is not clearly articulated. The authors should reorganize this discussion into a clearer cause-and-effect framework explaining how thermal and plasmonic effects act together.

3. The authors employ pulsed MIR excitation (Fig. 1b) while extracting thermal decay constants on microsecond timescales (Fig. 3e). The authors should clarify the motivation for using pulsed rather than continuous-wave MIR excitation and discuss whether CW excitation would yield equivalent results in the linear heating regime.

4. The extraction of effective molecular temperature (T_{mol}) from vibrational populations and electronic temperature (T_e) from electronic scattering (Section 5, SI) is critical in this study. The authors should provide additional methodological details regarding the fitting procedures, assumptions, calibration, and uncertainty associated with both temperature estimates. In addition, the discrepancy between the temperature changes derived from vibrational populations and those obtained from

the two-temperature model (line 150) should be explicitly discussed, particularly given that electron–phonon coupling timescales are expected to be much shorter than the microsecond time resolution of the experiment.

5. As shown in Table S1, most of the vibrational modes exhibit an MIR-induced temperature increase of approximately 70 K, whereas the mode at 331 cm^{-1} shows a significantly smaller increase (~30 K). The authors should explain the origin of this anomaly and whether this deviation arises from increased uncertainty in fitting due to enhanced electronic scattering background at low wavenumbers.

6. Both Stokes and anti-Stokes intensities change under MIR illumination (Fig. 1d and Fig. S3), but the analysis primarily focuses on changes in anti-Stokes intensity alone. The authors should justify this choice and clarify whether analysis based on the anti-Stokes to Stokes intensity ratio would provide a more robust temperature determination.

7. In Fig. 2b, the modulation induced by varying the visible probe power density does not appear to approach zero at low density. The authors should clarify the experimental conditions under which these measurements were performed, specify the reference used to compute the reported percentage changes, and provide an estimate of the associated measurement uncertainty, particularly at low excitation power densities.

8. In Fig. S1a, the SERS spectra of aggregated Au nanoparticles functionalized with NTP and CB[5] appear nearly identical, apart from a small wavenumber shift shown in the inset, despite the substantially different molecular structures of the two species. The authors should clarify whether this similarity would be expected. To support spectral assignment, the authors should perform bulk Raman measurements of NTP and CB[5] and compare them with the SERS spectra obtained from the nanogaps. Furthermore, since the nanogaps are proposed to contain a mixture of NTP and CB[5], characteristic vibrational modes from both molecules should be observable and identified.

9. In addition to thermal modulation of SERS intensities, a temperature gradient across the molecular junctions may generate a thermovoltage via the Seebeck effect, which could also influence the measured signal. The authors should discuss the possible contribution of thermoelectric effects and justify whether such contributions can be neglected under the present experimental conditions.

10. The stretched-exponential decay ($\beta < 1$) observed for mixed NTP/CB[5] nanogaps (Fig. 3e) is attributed to molecular impurity or compositional disorder. The authors should perform the same measurements on nanogaps containing only pure NTP or pure CB[5] to verify whether β approaches unity in a homogeneous system. Similarly, $\beta < 1$ is also observed for gold oxide nanogaps following O_2 plasma treatment (Fig. 4c), despite the claim that organic components are removed. This raises the possibility that residual inorganic heterogeneity or variations in intragap morphology contribute to the observed behaviour. The authors should provide direct structural evidence (e.g., S/TEM micrographs) to support this interpretation and expand the dataset beyond the two samples shown.

11. Some minor editorial points: (1) In Fig. 2c, the y-axis is missing unit, and the author should clarify whether the previously observed spectral shift of 5 cm^{-1} is a combined effect of the two lasers used; (2) In Line 189, Fig. 3d should be cited instead of Fig. 3b; (3) The 785 nm probe laser lies in the near infrared and should not be referred to as a visible laser throughout the manuscript as it sometimes causes confusion.

12. As an additional point, it would strengthen the generality of the conclusions if the authors could demonstrate, for singly thiolated molecules, the expected trend of decreasing thermal conductivity with increasing molecular length, analogous to the behaviour reported for dithiolated molecules.

Version 1:

Reviewer comments:

Reviewer #1

(Remarks to the Author)
The authors have not adequately addressed the criticisms in my first review. A measurement of the spot size dependence of the data is needed. The authors have not done this simple experiment to falsify my hypothesis about the mechanisms that govern the long time scales observed in these experiments.

Reviewer #2

(Remarks to the Author)
I believe authors have addressed reviewer's concerns adequately so the paper is now suitable for publication.

Reviewer #3

(Remarks to the Author)
I co-reviewed this manuscript with one of the reviewers who provided the listed reports. This is part of the Nature Communications initiative to facilitate training in peer review and to provide appropriate recognition for Early Career Researchers who co-review manuscripts.

Reviewer #4

(Remarks to the Author)
The authors provide generally clear and thoughtful responses to the reviewer's comments, particularly in clarifying the experimental methodology and expanding the discussion of thermal versus non-thermal effects. The additional details included in the revised manuscript and Supplementary Information significantly improve the transparency of the analysis and strengthen the reliability of the temperature extraction, especially through the more detailed treatment of uncertainties in T_{mol} and T_e. The quantitative consideration of the Seebeck effect is also appreciated and further supports the assignment of a predominantly thermal origin for the observed SERS modulation. Overall, these revisions address several of the concerns raised in the previous round of review and provide more robust support for the central conclusions. However, some aspects would still benefit from further clarification, particularly regarding the quantitative separation of thermal and plasmonic contributions, the robustness and consistency of the temperature extraction methods, and the interpretation of the stretched-exponential dynamics. Addressing these points would further strengthen the physical interpretation and overall scope of the study.
Comments:
1. The response and corresponding revisions regarding the exclusion of alternative mechanisms contributing to the anti-Stokes emission are generally well argued. In particular, the discussion based on characteristic timescales is reasonable and helps contextualize the role of pulsed excitation and observed decay dynamics. However, the argument concerning inelastic tunnelling remains somewhat less convincing in its current form. It would be beneficial if the authors could expand the discussion in the main text (e.g., in the section on MIR-induced temperature change) to more explicitly justify why the observed vibrational shifts can be attributed uniquely to a thermal origin. In particular, the manuscript would benefit from a clearer and more explicit comparison with alternative mechanisms, similar to the level of detail provided in the response letter.

2. In the previous revision, a concern was raised regarding the discrepancy between temperatures extracted from (i) vibrational populations and (ii) electronic Raman scattering. The authors attribute this discrepancy primarily to plasmonic enhancement effects. Given that this point is central to the interpretation of the results, it would be valuable to provide a more quantitative assessment of this effect. For example, incorporating information from dark-field scattering spectra and linking it to the calibration procedures described in Supplementary Section S5 would help substantiate this claim. At present, the explanation remains somewhat qualitative. A more quantitative treatment would clarify the reliability of the extracted temperatures. In this context, the temperature estimates derived from vibrational populations appear more robust, particularly given potential additional contributions to the SERS background (e.g., photoluminescence), which may affect the electronic scattering analysis.

3. The inclusion of additional TEM data is appreciated. While it is understood that experiments on purely NTP- or CB[5]-based systems are not feasible, the discussion of the stretched-exponential decay ($\beta < 1$) would benefit from further support. In particular, it would be helpful if the authors could provide additional statistical evidence (e.g., measurements across more samples or spatial positions) to support the assignment of $\beta < 1$ to molecular or structural disorder. Alternatively, establishing a correlation between the extracted β values and independent structural metrics (e.g. SEM/TEM-derived heterogeneity or variability in nanogap structure) would strengthen this interpretation.

Version 2:

Reviewer comments:

Reviewer #1

(Remarks to the Author)
The authors have adequately addressed the comments of my previous reviews. I recommend publication.

Open Access This Peer Review File is licensed under a Creative Commons Attribution 4.0 International License, which permits use, sharing, adaptation, distribution and reproduction in any medium or format, as long as you give appropriate credit to the original author(s) and the source, provide a link to the Creative Commons license, and indicate if changes were made.
In cases where reviewers are anonymous, credit should be given to 'Anonymous Referee' and the source.
The images or other third party material in this Peer Review File are included in the article’s Creative Commons license, unless indicated otherwise in a credit line to the material. If material is not included in the article’s Creative Commons license and your intended use is not permitted by statutory regulation or exceeds the permitted use, you will need to obtain permission directly from the copyright holder.
To view a copy of this license, visit https://creativecommons.org/licenses/by/4.0/

Response to reviewers:

We are delighted the reviewers find that our experiments "*conducted under precise control, and the analysis results are interesting*", and that "*time-resolved photothermal measurements with nanoscale precision in this manuscript would be of great interest*" (**Reviewer#2,3**), while presenting an "*innovative experimental approach for probing thermal transport*", "*carefully executed*", and "*represents a significant methodological advance with clear relevance*" (**Reviewer #4**). We note Reviewer #1 has perhaps misunderstood the key aspects of the experiment that the other reviewers appreciated. We deal with all their points below.

Reviewer #1:

The authors describe their experiments on thermal transport in 2D arrays of Au nanoparticles linked by organic molecules. I do not recommend publication. The authors are misinterpreting their experiments. The time-scale they observe for the temperature decay (on the order of 10 microseconds) is many orders of magnitude too long to be physically reasonable for the mechanism that they discuss. What I suspect is happening is that the 2D array of Au nanoparticles that is suspended over the 2 micron openings in the carbon grid is significantly heated by the 400 ns MIR laser pulse and then cools by lateral heat conduction and heat transfer into the carbon grid on a time scale governed by heat diffusion in the Au nanoparticle array on a length scale of 1 micron. Perhaps heat diffusion on the lateral scale of the MIR heating beam (4 micron 1/e^2 radius) is also involved. The authors could distinguish this mechanism from their proposed mechanism by changing the size of the holes in the carbon grid and changing the focus diameter of the MIR laser pulse.

The authors do not clearly explain what they think is happening to the temperature field in the samples. The authors are measuring a decay of the temperature of the molecules. I initially thought that the MIR beam is heating the molecules more than the Au nanoparticles and the authors were proposing that the molecules then cooled slowly into the adjacent Au, but the authors also state that changing the MIR wavelength gives the same result. That seems inconsistent with the discussion surrounding the cooling of the molecules into the Au nanoparticles. In any case, the cooling of molecules would be extremely fast, on a time scale <100 picoseconds, four orders of magnitude shorter than the time resolution of the measurement.

> It seems the reviewer is slightly confused but does eventually understand that our 2D array of Au nanoparticle is heated by the MIR pulse, and then cools by lateral conduction. What is the key aspect however is that this lateral conduction is not fast, because of the thermal barrier of molecules in each gap (which the other reviewers get is the interesting finding). So we do not look at molecular cooling per se (which is indeed fast), but how heat from hotter Au nanoparticles propagates through molecules to neighbours, which gives these much slower timescales. Indeed changing the array hole size will change the cooling rate as it changes the total thermal transport distance, but this is a trivial effect and it is changing the molecules that is the interesting aspect. While we measure the molecular temperature, we also show in Fig.1 that we measure the metal temperature also (and this agrees).

Reviewers #2 and #3:

1. It would be helpful to explain the main idea of how utilizing 2D NP sheets called MLagg can enable the measurement of thermal transport and what the relevant parameters are for the characterization of the nanostructures with more detailed definition of MLagg.

> Indeed this is a useful suggestion. The challenge so far for measuring thermal transport of molecular monolayers has been the difficulty of making a single precision nanogap and heating only one side rapidly (where light pulses are optimal). We avoid this by making many identical nanogaps, so it is easy to separate the heat source and the measurement. A key challenge has been making identical nanogaps, and this is why the MLagg sheet is an enabling advance. We now discuss this more clearly in the manuscript.

2. Please clarify the contradiction between the statement in line 65 and the Supplementary Section S2 (near-complete vs. partial replacement). Which one is correct? How do you conclude that these Raman and FT-IR show near-complete replacement of CB[5] into NTP throughout the sample?

> This is helpful. Clearly visible in Fig.S1b is that the original CB[5] lines almost completely disappear with NTP replacement. We estimate from these FTIR peaks that <10% CB[5] remains, for which both descriptions apply. We thus harmonised them to "near-complete", as suggested.

2. Please add references for the wavenumbers in lines 157, 159, and 160.

> These two vibrational resonances are assigned to the NTP nitro-stretch mode at ν_{NTP}=1335cm^{-1} and CH-stretch mode of CB[5] at ν_{CH}=1495cm^{-1}, from references [*J. Phys. Chem. C* **114**, 9 (2010); DOI:10.1021/jp911836k, *Nano Lett.* **22**, 17 (2022); DOI:10.1021/acs.nanolett.2c02806.]and [*Light Sci. Appl.* **11**, 281 (2022); DOI:10.1007/s10847-017-0751-3, *J. Incl. Phenom. Macrocycl. Chem.* **89** (2017); DOI:10.1038/s41377-022-00943-0] respectively. These have been added to the main text.

3. Since the authors characterized thermal energy transport based on the SERS technique, the distance between adjacent gold nanoparticles can be critical. Please provide the spectroscopic/microscopic data that can provide statistical information on the distance between gold nanoparticles, depending on the type of junctions.

> We have done extensive work over the past decade on the spacing produced by CB[5] molecules between Au NPs. Key early work uses spectroscopy of dimers and chains [ref 28] as well as the 'nanoparticle-on-mirror' [Faraday Discussions **178**, 185 (2015)] to measure gap sizes of d=0.9$\pm$0.05nm. Subsequent work on the CB-induced gaps in MLagg [refs 29,30] confirms this size in the MLagg sheets. We also have high-resolution TEM showing the same result (see **Fig.R1** below).

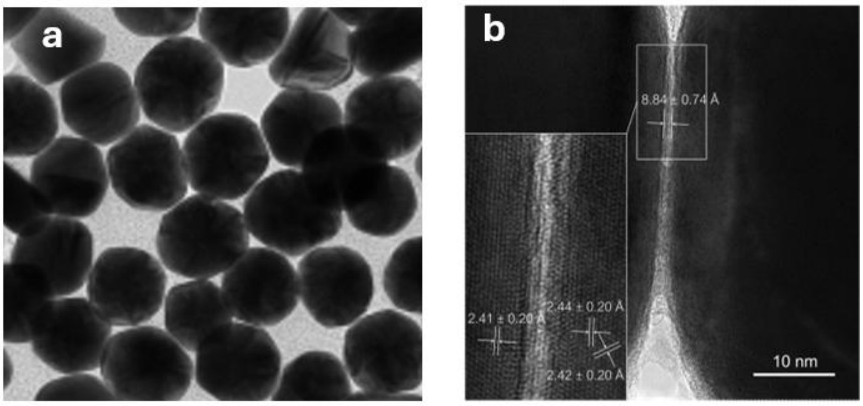

Fig.R1: **a**, TEM of free-standing MLagg sheet with CB[5] binding showing **b**, 0.9nm gap size.

4. Figure 2 discusses the frequency shift data, which is not explained in relation to the nanogaps or distance between the gold nanoparticles. It would be helpful to provide explanations about those effects on thermal energy transport.

> We believe the reviewer is asking if the observed frequency shift of the NO_2 group which agrees with the extracted heating from the SERS intensities, could lead to modified thermal transport. This is an interesting question, however for the ΔT=50-80K measured, little change in occupation of the vibrations and their frequency shift is expected (see reply to comment 2 of reviewer 4), therefore thermal transport is little changed. The thermal expansion of the gap is also negligible compared to the dynamical motion of the molecules (typically 10^{-5}/K [Chem. Soc. Rev. **49**, 3315 (2020); DOI 10.1039/C8CS00916C]).

5. The NEMD simulation section can be improved by correlating with experimental results. For example, it is not clear how authors know that the surface roughness of the gold contact leads to fewer fully-bridged Au-S-R-S-Au junction. Some explanations in the paragraph following this sentence are speculative, not substantiated by experimental results or prior results with references.

> We aimed to connect simulation and experiment as much as warranted. The experimental trends are well predicted but not their magnitudes, as we discuss. We agree that the Au nanoparticle surfaces in the experimental setup are not atomically flat. Consequently, a subset of the molecules fails to form bridges between both electrodes. To account for this surface roughness in our simulations, we modelled mixed BPT-BPDT samples (Fig. 5d), where a controlled fraction of molecules remains mono-thiolated and thus disconnected from one electrode. This approach allows us to maintain consistent force field potentials (specifically the Morse potential for flat surfaces) across all calculations, ensuring a rigorous and fair comparison. By introducing this mixture, we effectively reduce the degree of idealised contact, which leads to better agreement with the experimental data. This confirms that a perfectly bridged junction is an idealised case not fully realized in measurement. We have revised the manuscript to include a detailed discussion of these structural effects and have added a new reference [20] which similarly reports the overestimation of thermal conductance in perfectly constructed molecular contacts.

It is not possible to perform the computationally-heavy simulations with rough facets. However evidence for roughness reducing the number of contacts is known from conductivity measurements in molecular electronics [Appl. Phys. Lett. **120**, 112103 (2022); DOI 10.1063/5.0085778]. We agree that separation of evidence and discussion is helpful, and so explicitly do this now in the text.

6. In the conclusion section, it is not clear whether authors can claim that substrate conduction is eliminated as the spot size of MIR may be larger than the hole size of a-carbon grid, which is not discussed in detail. Also, it is not clear whether the technique can be claimed as an ultrasensitive photothermal SERS since the developed technique is for the characterization of sensitive heat conduction for the known molecule.

> These are useful points. Spatial measurements of the photothermal modulation (Fig.3d) show the effect essentially follows the MIR intensity profile which spans across several holes in the amorphous C grid. We now clearly indicate this in Fig.3d. The measured MLagg is thermally connected to areas in contact with the grid, however (unlike on a CaF_2 substrate), we still observe a modulated SERS signal. Any substrate conduction is therefore insignificant compared to the MLagg. FTIR measurements show minimal MIR absorption of the amorphous C grid (Fig. 3a). Each hole is thus heated by the power incident on it, and for the same number of gaps (since we use the same size nanoparticles) the resulting thermal decays then reflect the individual gap thermal conductivity. We agree this is helpful to clarify.

We also agree that describing this as a ‘photothermal’ technique is not the strongest aspect, and thus now amend as suggested to refer to the sensitive optical tracking of thermal conductivity.

7. Please improve writing and polish grammar for the overall manuscript, including:
- There should be space between numbers and units.
- Please separate materials and methods into another section to improve readability.

> We amended both as suggested.

Reviewer #4:

1. The authors report an increase in anti-Stokes SERS intensity upon MIR illumination (e.g., Fig. 1d and 2a) and attribute this primarily to an increased phonon population due to thermal excitation. However, similar increases in anti-Stokes emission have been observed in plasmonic systems arising from non-thermal mechanisms, such as molecular frequency up-conversion (e.g., Science 374, 1268–1271 (2021)), vibrational pumping, picocavity formation, inelastic tunnelling, and molecular optomechanical

effects. The authors should clarify how these alternative mechanisms are excluded in the present experiments and justify why the observed SERS modulation can be attributed predominantly to thermal effects.

> The reviewer cites many of our previous works, which we greatly appreciate. Indeed antiStokes emission can increase for a number of different reasons. The primary reason for identifying this as thermal is the rise and decay time-scales involved. MIR up-conversion disappears within a few ps, as does vibrational pumping and optomechanical effects, because the measured vibrational lifetimes are less than a few ps [DOI: 10.1103/PhysRevB.109.195404]. Inelastic tunnelling of electrons could only persist for μs if charge is redistributed by the MIR between nanoparticles and slowly tunnels back, but this would not explain the similar electronic heating observed, nor the vibrational shifts. Finally picocavity decay in these nanogaps is much slower, often >1s, and not linear in power as observed for the signals here. All these processes should also work for MLaggs on a CaF_2 substrate, in disagreement with our findings. We thus clarify the discussion as suggested.

2. The discussion of temperature-induced changes in Stokes vibrational and electronic scattering (In SI and Fig. 1,2) reads largely as a list of observations. While the authors dismiss ground-state depletion associated with increased vibrational population and instead emphasize plasmonic modulation, the physical relationship between temperature rise, plasmon resonance modulation, and the observed changes in SERS intensity is not clearly articulated. The authors should reorganize this discussion into a clearer cause-and-effect framework explaining how thermal and plasmonic effects act together.

> As reviewers #2,3 suggest, we try to separate observations from their subsequent discussion. However we agree that this can be improved. The reviewer focusses on the induced decrease in Stokes intensity. Depletion of ground-state population ($\propto 1 - \exp(-h\nu/kT)$) is neglected because the predicted change is only 0.5%, far less than the 30% observed. We thus refine this argument.

3. The authors employ pulsed MIR excitation (Fig. 1b) while extracting thermal decay constants on microsecond timescales (Fig. 3e). The authors should clarify the motivation for using pulsed rather than continuous-wave MIR excitation and discuss whether CW excitation would yield equivalent results in the linear heating regime.

> As long as the excitation is less than the μs decay times, the pulse length will not have any effect. Our motivation for using short pulses was to separately examine faster timescale effects.

4. The extraction of effective molecular temperature (T_{mol}) from vibrational populations and electronic temperature (T_e) from electronic scattering (Section 5, SI) is critical in this study. The authors should provide additional methodological details regarding the fitting procedures, assumptions, calibration, and uncertainty associated with both temperature estimates. In addition, the discrepancy between the temperature changes derived from vibrational populations and those obtained from the two-temperature model (line 150) should be explicitly discussed, particularly given that electron–phonon coupling timescales are expected to be much shorter than the microsecond time resolution of the experiment.

> It seems the reviewer may have missed that in the SI sections 5.2 and 5.3, we provided detailed methods for these extractions, including fitting methods. We now discuss the calibration and uncertainties in more detail. We agree that we would expect molecular and electronic temperatures to be the same and discuss why there might be a systematic difference in both the main text and SI. The difference is likely due to the relative plasmonic enhancement of antiStokes/Stokes frequencies which cannot be accurately removed from measurements and is shown to affect the photothermal modulation of measured s-MLaggs (Fig. S3).

5. As shown in Table S1, most of the vibrational modes exhibit an MIR-induced temperature increase of approximately 70 K, whereas the mode at 331 cm^{-1} shows a significantly smaller increase (~30 K). The authors should explain the origin of this anomaly and whether this deviation arises from increased uncertainty in fitting due to enhanced electronic scattering background at low wavenumbers.

> The values in Table S1 represent temperature estimates from one measurement used to calculate the average $\Delta\overline{\mathrm{T}}_{\mathrm{mol}}$ in Fig. 2d of the main text. We have clarified this is Supplementary Section S5.

6. Both Stokes and anti-Stokes intensities change under MIR illumination (Fig. 1d and Fig. S3), but the analysis primarily focuses on changes in anti-Stokes intensity alone. The authors should justify this choice and clarify whether analysis based on the anti-Stokes to Stokes intensity ratio would provide a more robust temperature determination.

> Because temperature changes manifest more clearly in the anti-Stokes (increasing from smaller signals), they are more suitable to use here. The discussion (see above) on the mechanism of Stokes changes makes it better not to use for comparing thermal conductivities, which is the main focus of this work.

7. In Fig. 2b, the modulation induced by varying the visible probe power density does not appear to approach zero at low density. The authors should clarify the experimental conditions under which these measurements were performed, specify the reference used to compute the reported percentage changes, and provide an estimate of the associated measurement uncertainty, particularly at low excitation power densities.

> The values in Fig. 2b represent the % intensity change with either MIR or 785nm power. While the 785nm power is varied, the MIR is kept constant at I_{MIR} = 50 μW/μm^2 and thus a non-zero modulation at all probe powers is seen. The modulation should not change at all with probe power, however, we suspect small heating by the probe itself is responsible for a slight change at lower powers.

8. In Fig. S1a, the SERS spectra of aggregated Au nanoparticles functionalized with NTP and CB[5] appear nearly identical, apart from a small wavenumber shift shown in the inset, despite the substantially different molecular structures of the two species. The authors should clarify whether this similarity would be expected. To support spectral assignment, the authors should perform bulk Raman measurements of NTP and CB[5] and compare them with the SERS spectra obtained from the nanogaps. Furthermore, since the nanogaps are proposed to contain a mixture of NTP and CB[5], characteristic vibrational modes from both molecules should be observable and identified.

> Possibly the reviewer did not appreciate <u>both</u> these samples have NTP in the nanogaps, but one was prepared initially with CB which was replaced, and the other was directly created with NTP. Indeed if they both produce identical NTP loadings with all CB replaced, then we expect them to be identical. This is also seen in the FTIR in Fig.S1b. The shift of the NO_2 line at 1335 cm^{-1} comes from its sensitivity to NTP proximity, showing the NTP density is slightly higher when preparing directly from NTP (as perhaps expected). We clarified the two samples now in Fig. S1a.

9. In addition to thermal modulation of SERS intensities, a temperature gradient across the molecular junctions may generate a thermovoltage via the Seebeck effect, which could also influence the measured signal. The authors should discuss the possible contribution of thermoelectric effects and justify whether such contributions can be neglected under the present experimental conditions.

> This is indeed an interesting point. The estimated ΔT ~50 K is defined relative to the temperature of the unperturbed s-MLagg. As the heating follows the MIR intensity profile (Fig.3d), this temperature gradient occurs over ~5 μm. For 60 nm AuNPs, this corresponds to a linear chain of ~80 nanogaps each experiencing much smaller local temperature gradients of ~0.5 K/gap. The Seebeck coefficient of single BDT molecules is about 10 μV/K, and the corresponding thermal voltage across nanogap

generates an electric field of approximately 2.5×10^3 V/m. While this gradient is non-trivial, it remains orders of magnitude weaker than the 10^7 to 10^8 V/m threshold typically required to induce observable vibrational Stark shifts in organic molecules [*Proc.Natl.Acad.Sci.* **111**, 4 (2014)]. We therefore conclude that the Seebeck-induced electric field is too weak to perturb the SERS intensities.

10. The stretched-exponential decay ($\beta < 1$) observed for mixed NTP/CB[5] nanogaps (Fig. 3e) is attributed to molecular impurity or compositional disorder. The authors should perform the same measurements on nanogaps containing only pure NTP or pure CB[5] to verify whether β approaches unity in a homogeneous system. Similarly, $\beta < 1$ is also observed for gold oxide nanogaps following O_2 plasma treatment (Fig. 4c), despite the claim that organic components are removed. This raises the possibility that residual inorganic heterogeneity or variations in intragap morphology contribute to the observed behaviour. The authors should provide direct structural evidence (e.g., S/TEM micrographs) to support this interpretation and expand the dataset beyond the two samples shown.
> Indeed this subtlety is interesting. Unfortunately it is not possible to make time-resolved measurements on a pure CB[5] sample, because the SERS signal is ~100x smaller (it has no π bonds). It is not possible to make measurements with pure NTP, because thiols bind to facets on both sides of the nanogap and do not properly assembly MLaggs. The gold oxide is an extremely sensitive system that we have studied elsewhere (see [30], now published Nature Chem. **18**, 294 (2026)) and shows disproportionation of different Au oxidation states. Much the best way to see if organics are all removed is the SERS, which shows that when properly carried out, no organics remain after oxidation (see [49]). We also provide TEM evidence, see above in **Fig.R1**.

11. Some minor editorial points: (1) In Fig. 2c, the y-axis is missing unit, and the author should clarify whether the previously observed spectral shift of 5 cm^{-1} is a combined effect of the two lasers used; (2) In Line 189, Fig. 3d should be cited instead of Fig. 3b; (3) The 785 nm probe laser lies in the near infrared and should not be referred to as a visible laser throughout the manuscript as it sometimes causes confusion.
> We thank the eagle-eyed reviewer and rectified all these mistakes.

12. As an additional point, it would strengthen the generality of the conclusions if the authors could demonstrate, for singly thiolated molecules, the expected trend of decreasing thermal conductivity with increasing molecular length, analogous to the behaviour reported for dithiolated molecules.
> Indeed this is a good idea. We concentrated here on di-thiols as they are known for molecular electronics and they span the gap. On the other hand, the complexity for monothiols is that their thermal conductivity is much worse (see BPT in Fig. 5c) without the non-thiolated end binding to the Au, and our simulations show conductivity is dominated by inter-molecular interactions. This is thus the subject of ongoing future work.

Response to reviewers:

We are delighted that reviewers 2 and 3 find we addressed their concerns and that the paper should be published. Reviewer 4 also commends our "*clarifying the experimental methodology and expanding the discussion of thermal versus non-thermal effects*", that we "*significantly improve the transparency of the analysis and strengthen the reliability of the temperature extraction*", and "*address several of the concerns raised in the previous round of review and provide more robust support for the central conclusions*". Reviewer 1 seems not to have noted our previous explanation, and we thus now show new data they request for a different spot size. All comments are addressed in full below.

Reviewer #1:

The authors have not adequately addressed the criticisms in my first review. A measurement of the spot size dependence of the data is needed. The authors have not done this simple experiment to falsify my hypothesis about the mechanisms that govern the long time scales observed in these experiments.

> The reviewer perhaps has not understood our response. They stated in their first review "Perhaps heat diffusion on the lateral scale of the MIR heating beam (4 micron 1/e^2 radius) is also involved". Indeed, as we tried to emphasise, this is exactly what is measured here. We now confirm this as requested, by taking new data with a different spot size. Since this is not simple when focussing a MIR beam through a Cassegrain reflecting objective, we compare to a 633nm visible laser which is focussed to a d~450nm spot (compared to the d~8µm spot of the MIR beam). Using this visible pump introduces complications from its own Stokes emission, but we are able to still resolve the 785nm antiStokes emission. We measure now an average decay time of 62±4ns for the BPT sample, compared to the 15µs seen with the MIR focus spot (**Fig.R1** below). This is exactly in line with the scaling expected [DOI: 10.1063/5.0019025] from the spot size ($\tau \propto d^2/\kappa$) which predicts 15µs x $(450/8000)^2$~60ns decay. The crucial point is that for fixed spot size, the decay is inversely proportional to the effective thermal conductivity of the MLagg. This is what allows us to directly compare conductivities for the different molecules. We agree that this is good to briefly discuss in the manuscript.

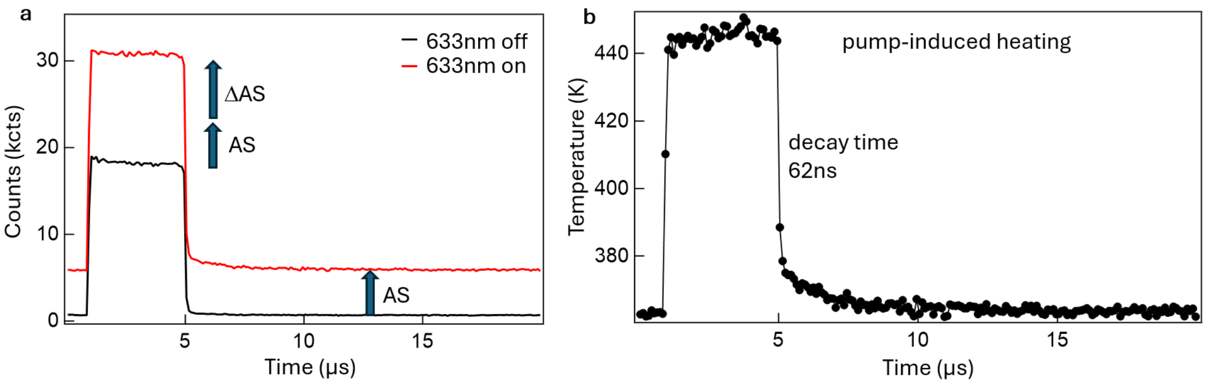

Fig.R1: a, Integrated anti-Stokes Raman signal at 1080cm^{-1} as a function of time, with 633nm pump on for 4µs both with (red) and without (black) the 785nm Raman probe. Arrows show the additional signal from the 785nm-induced antiStokes emission (AS) and the extra induced by the 633nm heating (ΔAS). **b,** Temperature extracted from difference in antiStokes signal, showing fast decay time.

Reviewer #2:

I believe authors have addressed reviewer's concerns adequately so the paper is now suitable for publication.

Reviewer #3:

I co-reviewed this manuscript with one of the reviewers who provided the listed reports.

Reviewer #4:

1. The response and corresponding revisions regarding the exclusion of alternative mechanisms contributing to the anti-Stokes emission are generally well argued. In particular, the discussion based on characteristic timescales is reasonable and helps contextualize the role of pulsed excitation and observed decay dynamics. However, the argument concerning inelastic tunnelling remains somewhat less convincing in its current form. It would be beneficial if the authors could expand the discussion in the main text (e.g., in the section on MIR-induced temperature change) to more explicitly justify why the observed vibrational shifts can be attributed uniquely to a thermal origin. In particular, the manuscript would benefit from a clearer and more explicit comparison with alternative mechanisms, similar to the level of detail provided in the response letter.

> We appreciate the encouragement to amplify our discussion in the main text, summarising alternative mechanisms and the evidence that points against them. The point about inelastic tunnelling by the reviewer is more interesting than we had realised. We have measured the electrical conductivity of free-standing MLaggs, which are in the 10^{-6}-10^{-7} S range, as well as previously examined the single junction conductance [Kos et al, *Quantum Tunneling…in Nanocavity Molecular Junctions*, ACS Nano (2021) DOI: 10.1021/acsnano.1c04100] which is in the 10^{-5}-10^{-4} S range for BPT. To evaluate the role of electrons in heat transport, we note that the electrical conductances of single molecules of PDT and BPDT are relatively low ($8x10^{-7}$ S and $3x10^{-7}$ S respectively) [Morikawa et al., Nanoscale 6, 8235 (2014); Guo et al., Nano Lett. 13, 4326 (2013)]. According to the Wiedemann-Franz law, these values yield an estimated electron thermal conductance of about 5pW/K for PDT and 2.5pW/K for BPDT. These electron contributions are orders of magnitude lower than the typical phonon thermal conductance in such systems [DOI 10.1021/acs.jpcc.8b12538, 10.1021/acs.nanolett.5c00225], so we conclude that the electron contribution to total heat transport is negligible. Heat dissipation in these junctions is thus primarily phonon-dominated. Furthermore, the agreement between the elastic phase-coherent phonon transport calculations and the single-molecule thermal conductance measurements [DOI 10.1021/acs.nanolett.9b02089] confirms that inelastic tunneling does not dominate the heat transport in molecules. This conclusion is also confirmed by the excellent agreement between our measurements and NEMD simulations shown in Figure 5. We now include this in our revised discussion, as suggested.

2. In the previous revision, a concern was raised regarding the discrepancy between temperatures extracted from (i) vibrational populations and (ii) electronic Raman scattering. The authors attribute this discrepancy primarily to plasmonic enhancement effects. Given that this point is central to the interpretation of the results, it would be valuable to provide a more quantitative assessment of this effect. For example, incorporating information from dark-field scattering spectra and linking it to the calibration procedures described in Supplementary Section S5 would help substantiate this claim. At present, the explanation remains somewhat qualitative. A more quantitative treatment would clarify the reliability of the extracted temperatures. In this context, the temperature estimates derived from vibrational populations appear more robust, particularly given potential additional contributions to the SERS background (e.g., photoluminescence), which may affect the electronic scattering analysis.

> Indeed as we mentioned in the previous response, the calibration of emission efficiencies modifies the effective temperatures extracted. Because the Stokes and antiStokes vibrational lines used are separated by wavelengths of up to 200nm, it is likely more accurate to use the electronic temperature where the fit uses a <30nm wavelength span. However we stress that this exact calibration is not important for the decays which characterise the different molecular thermal conductivities. While we have indeed tried to use dark-field scattering spectra, these measure far-field scattering and not the near-field emission efficiencies, which is why so far it has proved unfeasible [a detailed discussion can be found in DOI 10.1002/advs.202207178]. We thus feel it is most appropriate to provide both estimates, and point out the likely cause.

3. The inclusion of additional TEM data is appreciated. While it is understood that experiments on purely NTP- or CB[5]-based systems are not feasible, the discussion of the stretched-exponential decay ($\beta < 1$) would benefit from further support. In particular, it would be helpful if the authors could provide additional statistical evidence (e.g., measurements across more samples or spatial positions) to support the assignment of $\beta < 1$ to molecular or structural disorder. Alternatively, establishing a correlation between the extracted β values and independent structural metrics (e.g. SEM/TEM-derived heterogeneity or variability in nanogap structure) would strengthen this interpretation.

> The reviewer notes indeed an interesting aspect of our work, however we emphasise that the $\beta<1$ analysis forms a very limited part of this work, pointed out for interest in a few sentences, rather than central to any key argument here (which instead uses $\beta=1$ data). Indeed we have measured at different points on several samples and find the results are consistent. While it is not appropriate to further expand on the influence of disorder here, we note new experiments are currently underway to examine this difficult regime to carefully correlate disorder and thermal decay rates.
